# Characterization and engineering of branched short-chain dicarboxylate metabolism in *Pseudomonas* reveals resistance to fungal 2-hydroxyparaconate

**DOI:** 10.1016/j.ymben.2022.12.008

**Published:** 2023-01

**Authors:** Jan de Witt, Philipp Ernst, Jochem Gätgens, Stephan Noack, Davina Hiller, Benedikt Wynands, Nick Wierckx

**Affiliations:** a Institute of Bio- and Geosciences IBG-1: Biotechnology, Forschungszentrum Jülich, Jülich, Germany; b Institut für Mikrobiologie, Technische Universität Braunschweig, Germany

**Keywords:** Itaconate, Metabolic engineering, *Pseudomonas putida* KT2440, 2-hydroxyparaconate, Plastic-upcycling, *Pseudomonas aeruginosa* PAO1

## Abstract

In recent years branched short-chain dicarboxylates (BSCD) such as itaconic acid gained increasing interest in both medicine and biotechnology. Their use as building blocks for plastics urges for developing microbial upcycling strategies to provide sustainable end-of-life solutions. Furthermore, many BSCD exhibit anti-bacterial properties or exert immunomodulatory effects in macrophages, indicating a medical relevance for this group of molecules. For both of these applications, a detailed understanding of the microbial metabolism of these compounds is essential. In this study, the metabolic pathway of BSCD degradation from *Pseudomonas aeruginosa* PAO1 was studied in detail by heterologously transferring it to *Pseudomonas putida*. Heterologous expression of the PA0878-0886 itaconate metabolism gene cluster enabled *P. putida* KT2440 to metabolize itaconate, (*S*)- and (*R*)-methylsuccinate, (*S*)-citramalate, and mesaconate. The functions of the so far uncharacterized genes PA0879 and PA0881 were revealed and proven to extend the substrate range of the core degradation pathway. Furthermore, the uncharacterized gene PA0880 was discovered to encode a 2-hydroxyparaconate (2-HP) lactonase that catalyzes the cleavage of the itaconate derivative 2-HP to itatartarate. Interestingly, 2-HP was found to inhibit growth of the engineered *P. putida* on itaconate. All in all, this study extends the substrate range of *P. putida* to include BSCD for bio-upcycling of high-performance polymers, and also identifies 2-HP as promising candidate for anti-microbial applications.

## Highlights

• Engineered branched short-chain dicarboxylate (BSCD) metabolism in *P. putida*.
• Characterized transport and substrate range of BSCD pathway from *P. aeruginosa*.
• Revealed a link to fungal secondary metabolite resistance to 2-hydroxyparaconate.
• Engineered *P. putida* enables bio-upcycling of BSCD-containing polymer hydrolysates.

## Graphical abstract

**Figure:**
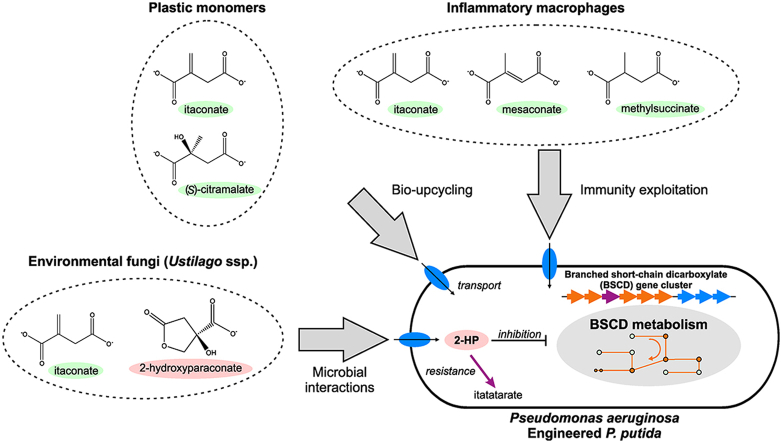

## 1 Introduction

The global plastic crisis urges us to redesign the entire life cycle of polymers. Nearly 400 Mt of plastics were produced in 2020 (Hundertmark et al., 2018), and estimated 90 Mt of plastic waste is predicted to enter the aquatic ecosystems by 2030 (Borrelle et al., 2020). This underlines the need for a more sustainable plastics life cycle. Ideally, polymers should be designed to be both bio-based and biodegradable to show a high degree of sustainability while also reducing the environmental burden (Wei et al., 2020). In recent years, major progress was achieved in the (bio-) catalytic depolymerization of plastics including polyesters, polyurethanes, and polyamides (Ellis et al., 2021; Magnin et al., 2020; Negoro et al., 2018; Tournier et al., 2020). Due to the heterogenic composition of many plastic products, their depolymerization results in a diverse mixture of monomeric building blocks. To close the life cycle of these plastics, the obtained mixed plastic hydrolysates need to be re- or upcycled. For the latter, microbial upcycling can be a powerful tool enabling the conversion of mixed plastic hydrolysates to value-added compounds (Tiso et al., 2021; Wierckx et al., 2015). Microbial upcycling requires efficient funneling of plastic monomers into the central metabolism of suitable microorganisms. *Pseudomonas putida* KT2440 and related species are well known in this respect, and strains have been isolated or engineered to funnel prevalent plastic monomers such as ethylene glycol (Franden et al., 2018; Li et al., 2019), 1,4-butanediol (Li et al., 2020), adipate (Ackermann et al., 2021), terephthalate (Narancic et al., 2021), and 2,4-toluenediamide (Puiggené et al., 2022) into their central metabolism. Furthermore, non-pathogenic Pseudomonads enable safe and practical research and were also engineered to produce a variety of value-added compounds, including polyhydroxyalkanoates (PHAs) (Dalton et al., 2022; Mezzina et al., 2021), aromatic compounds (Schwanemann et al., 2020) as well as rhamnolipids (Tiso et al., 2020). The direct upcycling of plastic monomers to such products was also achieved by engineering non-pathogenic Pseudomonads (Kenny et al., 2012; Werner et al., 2021). Recently, a two-stage chemical oxidation and biological funneling approach using engineered *P. putida* was developed enabling upcycling of various plastics (Sullivan et al., 2022). Application of this method on polypropylene (PP) would result in the release of branched short-chain dicarboxylates (BSCD).

Aliphatic dicarboxylates are widely used for the synthesis of polyesters, polyamides and polyurethanes. In the past years, the subgroup of BSCD gained importance as bio-based platform chemicals (Klement and Büchs, 2013; Saha, 2017; Üzüm and Karadağ, 2006; Wu et al., 2021; Xie et al., 2014). Especially their use as bio-based monomers for plastics is of great interest, as certain branches allow cross-linking of polymer chains and polymer properties can be tuned (Little et al., 2020; Voit and Lederer, 2009; Xie et al., 2014). Among BSCD, itaconic acid is the most prominent platform chemical as it can be used for plastic production, water decontamination, controlled drug delivery systems and many more (Okabe et al., 2009; Steiger et al., 2017; Willke and Vorlop, 2001).

Besides its use as bio-based platform chemical, itaconate was revealed as mammalian metabolite synthesized by the product of the immunoresponsive gene 1 (*irg1*) upon macrophage activation (Michelucci et al., 2013; Strelko et al., 2011). Itaconate not only triggers anti-inflammatory cascades (Mills et al., 2018) but also possesses anti-bacterial properties by inhibiting the isocitrate lyase that is the key enzyme of the glyoxylate cycle (Hillier and Charnetzky, 1981; Höner Zu Bentrup et al., 1999). Since the glyoxylate cycle is essential for pathogenic bacteria to survive in the host environment, itaconate inhibits growth of pathogens (Hillier and Charnetzky, 1981).

Nowadays, itaconate can be produced by fermentation processes from renewable feedstocks using *Aspergillus terreus* and species of the genus *Ustilaginaceae* such as *Ustilago maydis* and *Ustilago cynodontis* (Becker et al., 2021; Geiser et al., 2014; Guevarra and Tabuchi, 1990; Okabe et al., 2009; Wierckx et al., 2020). Besides itaconate, species of the genus *Ustilago* also produce two derivatives, namely its chiral lactone 2-hydroxyparaconate (2-HP) and itatartarate (ITT) (Guevarra and Tabuchi, 1990). In *U. maydis*, itaconate is converted to 2-HP by the cytochrome P450 monooxygenase Cyp3 (Geiser et al., 2016a). It is anticipated that a putative ring-cleaving dioxygenase (Rdo1) converts 2-HP to ITT, although this function could thus far not be confirmed (Geiser et al., 2016a, 2018). Both molecules, 2-HP and ITT, cannot be synthesized as pure compounds yet and are not commercially available, but they might exhibit interesting physiological properties due to their metabolic linkage to itaconate.

Many pathogens such as *Salmonella typhimurium*, *Yersinia pestis*, and *Pseudomonas aeruginosa* PAO1 have acquired an itaconate degradation pathway to overcome the anti-bacterial effects of itaconate (Martin et al., 1961; Sasikaran et al., 2014) (Fig. 3). Most itaconate-degrading pathogens such as *Y. pestis* contain an operon only encoding an itaconyl-CoA transferase (Ict), itaconyl-CoA isomerase/mesaconyl-C_4_-CoA hydratase (Ich), and (*S*)-citramalyl-CoA lyase (Ccl) (Sasikaran et al., 2014). In contrast, in the genome of *P. aeruginosa* PAO1 these enzymes are encoded in the PA0878-PA0883 operon which contains three additional genes of thus far unknown functions. The combination of these three genes is relatively specific to *P. aeruginosa*, although some species contain one or two similar genes (Sasikaran et al., 2014).

The interest in BSCD as bio-based polymer building blocks is rising. Furthermore, oxidative depolymerization of prevalent plastics such as PP results in the formation of BSCD (Partenheimer, 2003; Sullivan et al., 2022). To close the life cycle of such plastics, microbial upcycling using *P. putida* KT2440 could be applied. So far, itaconate degradation seems to be limited to pathogenic species that are not suitable for biotechnological applications (Sasikaran et al., 2014). Furthermore, the degradation of other BSCD such as (*S*)-(*R*)-methylsuccinate is not fully unraveled yet. Besides their usage as bio-based building blocks, many BSCD such as itaconate, mesaconate or citraconate were recently identified as anti-bacterial and immunomodulatory compounds (Bernard, 2022; Chen et al., 2022; He et al., 2022; McGettrick and O’Neill, 2022). Hence, the identification and characterization of bacterial metabolic pathways of BSCD is also of great importance to combat pathogenic and multidrug-resistant species.

In this study, the metabolic pathways for itaconate and other BSCD from *P. aeruginosa* PAO1 were characterized through their heterologous expression in the non-pathogenic *P. putida* KT2440. Peripheral degradation pathways for various BSCD were elucidated and a general BSCD uptake transporter was identified. Metabolic engineering enabled efficient growth of *P. putida* KT2440 on 20 mM of different BSCD within 16 h. Hence, a metabolic pathway of notable medical relevance was characterized, and at the same time, the range of upcyclable plastic monomers by *P. putida* KT2440 is extended. Furthermore, the functions of three so-far uncharacterized genes from the PA0878-0883 itaconate degradation cluster were explored and linked to the degradation of the itaconate derivatives 2-HP and ITT which have anti-bacterial and anti-fungal properties and interfere with the ability of bacteria to grow on itaconate.

## 2 Results and discussion

### 2.1 Degradation and transport of BSCD by engineered *P. putida* KT2440

*In vitro* studies indicated that degradation of itaconate requires the enzymes Ict, Ich, and Ccl that are encoded in the PA0878*-*PA0883 gene cluster of *P. aeruginosa* PAO1 (Sasikaran et al., 2014). Homologs of the encoding genes occur almost exclusively in pathogenic bacteria, whereas non-pathogenic strains such as *P. putida* KT2440 lack these genes. This highlights the importance of itaconate degradation by pathogens to survive in the host environment (Cordes and Metallo, 2021). Since Ict of *P. aeruginosa* PAO1 showed activity not only towards itaconate but also (*S*)-citramalate and methylsuccinate (Sasikaran et al., 2014), the PA0878-PA0883 gene cluster might encode degradation pathways for further BSCD. Especially the uncharacterized genes PA0879, PA0880, and PA0881 might be relevant for funneling additional BSCD into the pathway.

**Fig. 1:**
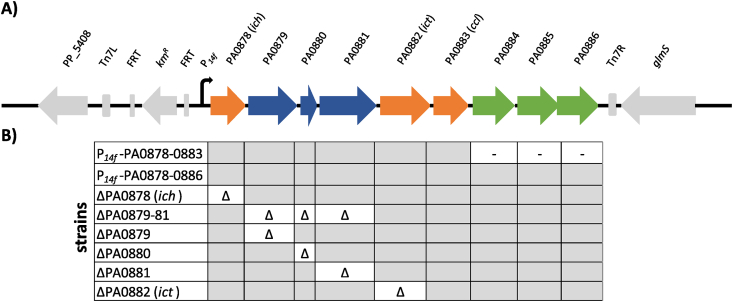
**Heterologous expression of the PA0878**–**0886 cluster in *P. putida* KT2440. (A)** The PA0878-PA0883 and PA0878-PA0886 clusters of *P. aeruginosa* PAO1 were fused to the strong constitutive *P*_*14f*_ promoter and integrated into the *attTn7* site of *P. putida* KT2440. PA0878 (*ich*), PA0882 (*ict*) and PA0883 (*ccl*) (orange) are involved in itaconate degradation. PA0879, PA0880 and PA0881 encode a putative acyl-CoA dehydrogenase, a putative ring-cleaving dioxygenase and an MmgE/PrpD family protein (blue). A putative BSCD transporter is encoded by PA0884-PA0886 (green). **(B)** Overview of investigated strains in this study. Grey indicates the presence of a gene, whereas absent and deleted genes are marked with a “-” and “Δ”, respectively.

To investigate the metabolic functions of the PA0878-PA0883 gene cluster from *P. aeruginosa* PAO1, it was fused to the strong constitutive *P*_*14f*_ promoter (Zobel et al., 2015) and chromosomally integrated into the *Tn7* attachment site in the genome of *P. putida* KT2440 (Fig. 1A). Additional genes (PA0884-PA0886) encoding a putative dicarboxylate transporter were identified 112 bp downstream of PA0883. Using the operon-mapper (Taboada et al., 2018), it was predicted that both gene clusters are encoded in a single PA0878-PA0886 operon. To test whether the putative transporter is involved in the degradation of BSCD, a second construct with the entire PA0878-PA0886 gene cluster fused to *P*_*14f*_ was integrated as well (Fig. 1B). The resulting strains were grown in minimal medium supplemented with 20 mM of the following BSCD as sole carbon source: itaconate, (*S*)-citramalate, (*R*)-citramalate, mesaconate, citraconate, and racemic (*S*)-(*R*)-methylsuccinate.

**Fig. 2:**
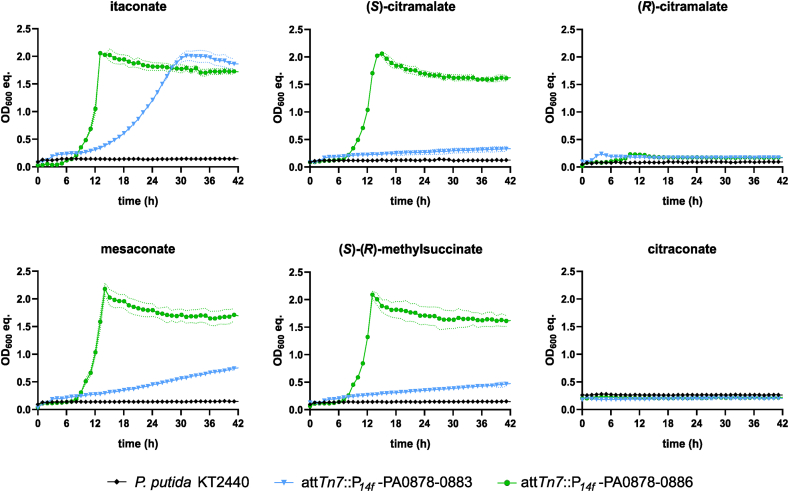
Substrate range of *P. putida* KT2440 strains harboring the PA0878-0883 cluster and effect of the PA0884-0886 BSCD transporter. *P. putida* KT2440 wild-type and the two strains *attTn7*:*P*_*14f*_ -PA0878-0883 and *attTn7*:*P*_*14f*_ -PA0878-0886 were grown in a Growth Profiler in 96-well microtiter plates with minimal medium containing 20 mM of the indicated BSCD as sole carbon source. OD_600_ equivalents (OD_600_ eq.) were derived from green-values obtained from the Growth Profiler using a calibration curve. Growth rates are shown in Table S1. The mean values with standard deviation (SD) of three replicates are shown.

Wild-type *P. putida* KT2440 was not able to utilize any of the tested substrates (Fig. 2). Integration of the PA0878-PA0883 cluster enabled *P. putida* KT2440 to grow on itaconate, (*S*)-citramalate, mesaconate, and (*S*)-(*R*)-methylsuccinate as sole carbon source, whereas (*R*)-citramalate and citraconate were not metabolized. However, growth on most BSCD was relatively slow (ranging from 0.11 ± 0.00 h^−1^ for itaconate to 0.01 ± 0.00 h^−1^ for citramalate) compared to typical growth on glucose or other plastic monomers (Ackermann et al., 2021; Li et al., 2020). Inclusion of the putative transporter-encoding genes in the *P*_*14f*_ -PA0878-PA0886 construct enabled much faster growth rates around 0.4 h^−1^ (Fig. 2, Table S1). Thus, PA0884-PA0886 likely encodes a BSCD transporter importing itaconate, mesaconate, (*S*)-citramalate, and (*S*)-(*R*)-methylsuccinate, making it a dedicated uptake system for these compounds in *P. aeruginosa*. Furthermore, these results indicate the presence of a native transporter in *P. putida* KT2440 that facilitates uptake of itaconate at a moderate rate, and uptake of (*S*)-citramalate, mesaconate and (*S*)-(*R*)-methylsuccinate at a minor rate. Homologs of the BSCD transporter were identified in several *Pseudomonas* species that harbor a homologous six-gene itaconate metabolism cluster. Thus, the identified transporter might be a potential drug target for treatment of these pathogens using transport inhibitors. Interestingly, no homologous transporter system was identified in genetic vicinity of the three-gene operons of other pathogens such as *Y. pestis* and *Mycobacterium tuberculosis.* Instead, transporters with low similarities to PA0884-0886 were found in these strains, not associated with the itaconate metabolic operon (sequence identity <35%, query coverage <40%, E-value cutoff e^−20^). Hence, itaconate uptake within these species could be investigated in future studies to identify alternative transporters.

### 2.2 Characterization of peripheral BSCD pathways

**Fig. 3:**
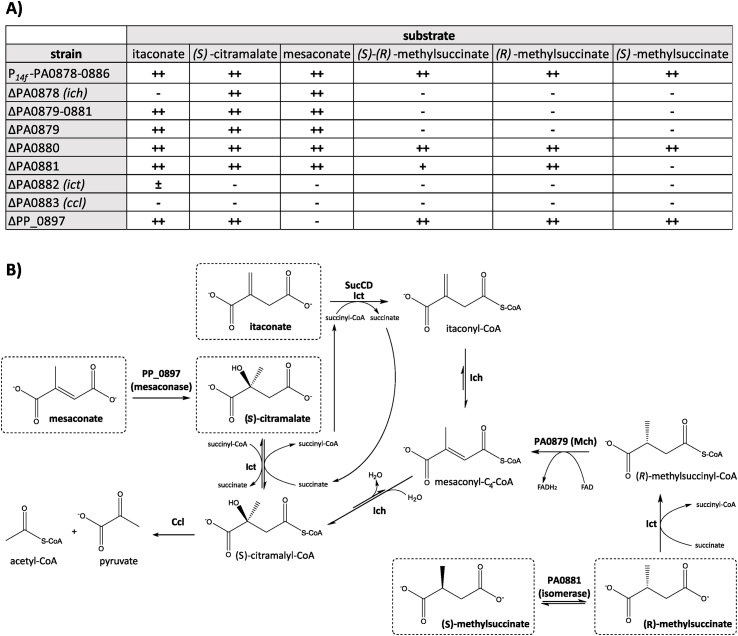
Proposed degradation pathways of BSCD encoded by the PA0878-0886 gene cluster. (A) *P. putida* KT2440 *attTn7*:*P*_*14f*_-PA0878-0886 and its deletion mutants were grown on the indicated BSCD as sole carbon source. Growth of the mutants was classified into the following categories: Final OD_600_ of >1.5 is reached within16 h (++), final OD_600_ of <1.5 reached within 16 h (+), final OD_600_ of >1 reached after 36 h (±), and no growth (−). Associated growth curves are shown in Fig. S1 and growth rates are displayed in Table S1 (B) Degradation pathways of the five BSCD (dashed boxes).

To further investigate the individual degradation pathways for each BSCD, different mutants were designed based on *P. putida* KT2440 *attTn7::P*_*14f*_-PA0878-PA0886 lacking individual genes of the expression cassette (Fig. 1). These constructs also allow to study a putative involvement of the uncharacterized genes PA0879, PA0880, and PA0881 encoding a putative acyl-CoA dehydrogenase, a probable ring-cleaving dioxygenase and an MmgE/PrpD family protein, in these pathways. Efficient growth on itaconate required the expression of *ich*, *ict*, and *ccl* whereas it was not affected by the deletion of PA0879, PA0880, and PA0881 (Fig. 3A). Although strain ΔPA0882 (Δ*ict*) could grow on itaconate, this growth was delayed, and slower (μ = 0.11 ± 0.00 h^−1^) compared to the strain expressing the full operon, which is likely due to the moonlighting activity of succinyl-CoA synthetase (SucCD) towards itaconate (Sasikaran et al., 2014). These results verify the previously proposed degradation pathway of itaconate based on *in vitro* assays *via* CoA-activation (by Ict) followed by isomerization/hydration (by Ich), and cleavage into acetyl-CoA and pyruvate (by Ccl) (Sasikaran et al., 2014) and *P. aeruginosa* PAO1 Δ*ict* experiments (Riquelme et al., 2020) (Fig. 3B). In contrast to growth on itaconate, the absence of Ict abolished growth on (*S*)-citramalate, suggesting that this substrate is not recognized by SucCD. Degradation of (*S*)-citramalate only required the expression of *ict* and *ccl*, indicating the direct conversion of (*S*)-citramalate to (*S*)-citramalyl-CoA followed by cleavage to acetyl-CoA and pyruvate (Fig. 3B). In contrast, (*R*)-citramalate was not metabolized. These results support the previously described stereoselectivity of Ict (Sasikaran et al., 2014). Degradation of mesaconate also required the presence of Ict and Ccl, indicating a similar degradation pathway as for (*S*)-citramalate (Fig. 3A). Neither Ich, nor any of the uncharacterized proteins were required for mesaconate degradation. Thus, mesaconate is likely not directly CoA-activated by Ict to mesaconyl-C_4_-CoA, since that would require Ich for further degradation. In *Burkholderia xenovorans*, Bxe_A3136 encodes a class I fumarase catalyzing the hydration of mesaconate to (*S*)-citramalate, thus enabling growth on mesaconate *via* Ict and Ccl (Kronen et al., 2015). The PP_0897 enzyme encoded in the genome of *P. putida* KT2440 shares a sequence identity of 75% with Bxe_A3136 at the protein level, making it a likely homolog. This was confirmed by deletion of PP_0897 in *P. putida* KT2440 *attTn7::P*_*14f*_ -PA0878-PA0886, which abolished growth on mesaconate (Fig. 3A, Fig. S1). Furthermore, (*S*)-citramalate accumulated in culture supernatants when the Δ*ict* mutant was grown with mesaconate and glucose as mixed substrates (Fig. S2).Thus, PP_0897 was revealed as mesaconase hydrating mesaconate to (*S*)-citramalate, which is further metabolized as described above (Fig. 3B). Furthermore, the ΔPP_0897 mutant showed reduced growth on acetate (data not shown), indicating a role as fumarase of the encoded enzyme in the TCA cycle hydrating fumarate to (*S*)-malate.

Besides Ict, Ich, and Ccl, growth on (*S*)-(*R*)-methylsuccinate also required the presence of the PA0879 gene, indicating that the corresponding product has methylsuccinyl-CoA dehydrogenase activity (Fig. 3A). Interestingly, the absence of the MmgE/PrpD family protein encoded by PA0881 led to reduced growth with the racemic substrate (μ = 0.18 ± 0.00 h^−1^) (Fig. 3A). Since half of the optical density was reached for the ΔPA0881 mutant (Fig. S1), PA0881 is likely involved in the metabolism of one enantiomeric form of methylsuccinate. To elucidate which enantiomer is degraded *via* the MmgE/PrpD family protein, growth was analyzed with either (*S*)- or (*R*)-methylsuccinate as pure substrate. Indeed, (*R*)-methylsuccinate was degraded in the absence of PA0881, whereas degradation of the (*S*)-enantiomer required the presence of PA0881 (Fig. 3A). Based on these results it can be concluded that (*R*)-methylsuccinate is CoA-activated by Ict to (*R*)-methylsuccinyl-CoA, which is further converted to mesaconyl-C_4_-CoA by the identified methylsuccinyl-CoA dehydrogenase (Mch) encoded by PA0879. Formation of mesaconyl-C_4_-CoA as intermediate is highly likely to occur, because the presence of Ich was still required for (*R*)-methylsuccinate metabolism. Utilization of (*S*)-methylsuccinate additionally required the presence of PA0881 indicating that at least one enzyme of the (*R*)-methylsuccinate degradation pathway is enantioselective for the (*R*)-enantiomer. Ict was previously revealed to be enantioselective towards (*S*)-citramalate and not (*R*)-citramalate (Sasikaran et al., 2014). Thus, activity of Ict might also be limited towards (*R*)-methylsuccinate. This is supported by the fact that the spatial position of the methyl group in relation to the C_4_ atom, where the CoA-activation takes place, is the same for (*S*)-citramalate and (*R*)-methylsuccinate (Fig. 3B). Thus, the PA0881-encoded MmgE/PrpD family protein likely acts as an (*S*)-(*R*)-methylsuccinate isomerase converting the (*S*)- to the (*R*)-enantiomer. Alternatively, PA0881 might also be an (*S*)-(*R*)-methylsuccinyl-CoA isomerase. Since Mch acts near the chiral center of (*R*)-methylsuccinyl-CoA this enzyme could in principle also be enantioselective for (*R*)-methylsuccinyl-CoA. For human very long-, long- and medium-chain acyl-CoA dehydrogenases an enantioselectivity was revealed as they only acted on the respective (*S*)-2-methylacyl-CoA-enantiomer (Battaile et al., 1998). In contrast to this, a human short/branched-chain acyl-CoA dehydrogenase was found to be active on both (*S*)- and (*R*)-2-methylbutyryl-CoA despite showing a preference for the (*S*)-enantiomer (Korman et al., 2005; Vockley et al., 2000).

The MmgE/PrpD family (IPR005656) is a rather heterogeneous family with members exhibiting diverse metabolic functions. Most members of this family are annotated as 2-methylcitrate dehydratase (EC 4.2.1.79) (PrpD) that are involved in propionate catabolism, catalyzing the third step of the 2-methylcitric acid cycle (Rocco et al., 2017). Moreover, members of the MmgE/PrpD family share high sequence identities with *cis*-aconitate decarboxylases (CAD) that convert *cis*-aconitate into itaconate (EC 4.1.1.6) . However, no dehydration or decarboxylation step is required for funneling (*S*)-methylsuccinate into the revealed pathway for its (*R*)-enantiomer. A unique function within the MmgE/PrpD family was revealed for an iminodisuccinate (IDS) epimerase from *Agrobacterium tumefaciens* BY6. This enzyme catalyzes the epimerization of (*R*),(*R*)-, (*S*),(*S*)- and (*R*),(*S*)-IDS and represents the only isomerase within the MmgE/PrpD family, so far (Lohkamp et al., 2006). To gain a detailed insight and to locate the PA0881-encoded protein in this functionally diverse protein family, ColabFold was used to predict its protein structure (Mirdita et al., 2021) (Fig. S3). Distance Matrix Alignment (DALI) was used to align the predicted structure to entries of the Protein Data Bank (PDB) to perform a structure-based identity search (Holm, 2020). Using this approach, it was revealed that the PA0881-encoded MmgE/PrpD family protein shared the highest structure identity with the IDS epimerase from *A. tumefaciens* BY6 (Table S2). Taken together, the structural similarity to the IDS epimerase, the probable enantioselectivity of Ict towards (*R*)-methylsuccinate, the fact that most well-studied MmgE/PrpD family proteins act on non-CoA-activated molecules, and the experimental results, all strongly indicate that the PA0881 MmgE/PrpD family protein acts as (*S*)-(*R*)-methylsuccinate isomerase to enable growth on (*S*)-methylsuccinate.

### 2.3 Degradation of itaconate in the presence of its derivatives (*S*)-2-hydroxyparaconate and itatartarate

The putative ring-cleaving dioxygenase encoded by PA0880 (hence named Rdo_PA_) is the last protein with unknown function encoded within the PA0878-0886 operon. With a size of 127 amino acids, Rdo_PA_ is a relatively small protein. Interestingly, Rdo_PA_ has a protein sequence identity of 59.7% compared to the Rdo1 putative ring-cleaving dioxygenase encoded in the itaconate production gene cluster of *U. maydis* (Geiser et al., 2016b). In *U. maydis* itaconate is further converted to its chiral lactone (*S*)-2-hydroxyparaconate (2-HP) by the cytochrome P450 monooxygenase Cyp3 (Geiser et al., 2016a). Permeabilized cells of *U. cynodontis* convert 2-HP to itatartarate (ITT) (Guevarra and Tabuchi, 1990), and it is anticipated that Rdo1 catalyzes this conversion although this could thus far not be confirmed (Geiser et al., 2016a, 2018). Both compounds might exhibit interesting physiological properties due to their metabolic linkage to itaconate, and we hypothesized that Rdo_PA_ could be involved in their degradation due to the sequence similar to Rdo1. Since 2-HP and ITT are not commercially available, the engineered *Ustilago cynodontis* NRBC 9727 Δ*fuz7* (Hosseinpour Tehrani et al., 2019), was used to produce a mixture of itaconate, 2-HP, and ITT. The obtained mixture was diluted to contain 20 mM of itaconate, adjusted to pH of 6.7, and was used to investigate the degradation of itaconate in the presence of 2-HP and ITT as well as the putative function of Rdo_PA_ as 2-HP lactonase.

**Fig. 4:**
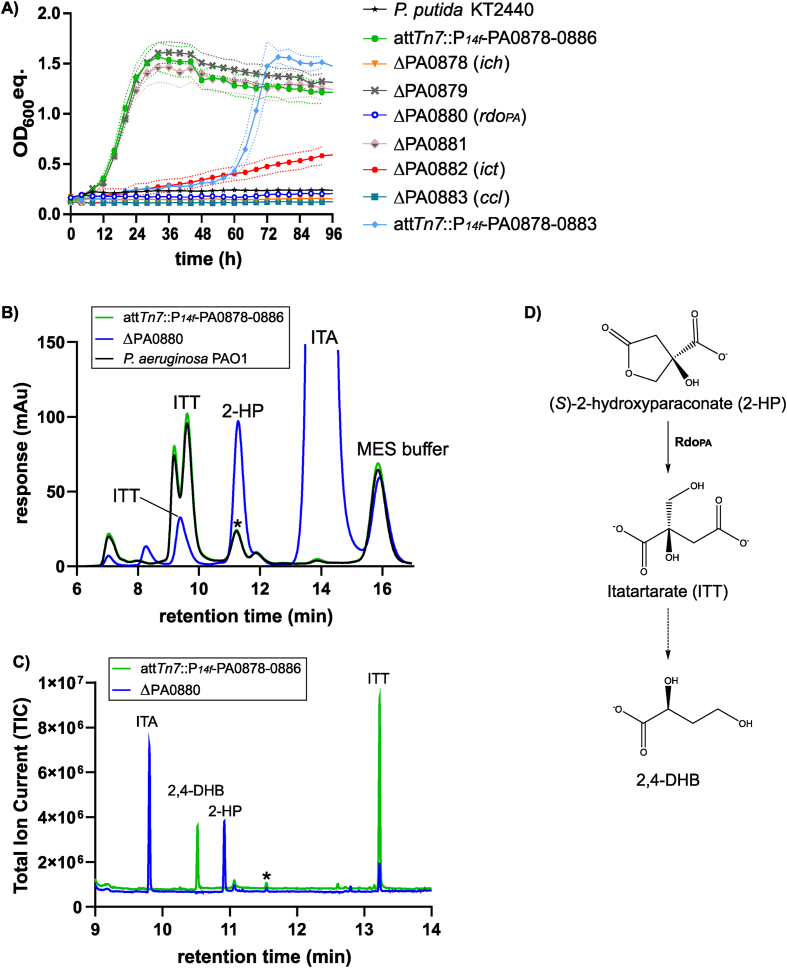
Effects of 2-HP and ITT on itaconate metabolism. (A) Strains of *P. putida* KT2440 were grown in a Growth Profiler in 96-well microtiter plates with minimal medium containing a mixture of itaconate, 2-hydroxyparaconate (2-HP) and itatartarate (ITT). The mixture was diluted to contain 20 mM of itaconate, whereas concentrations of 2-HP and ITT were approximately 32 mM and 17 mM. OD_600_ equivalents (OD_600_ eq.) were derived from green-values obtained from the Growth Profiler using a calibration curve. Growth rates are shown in Table S1. The mean and standard deviation (SD) of three replicates is shown. (B) HPLC chromatogram of samples obtained from cultivation of indicated strains with the itaconate/2-HP/ITT mixture after 72 h. Response of the DAD signal (λ = 210 nm) is shown in arbitrary units (mAu). The peak shift of ITT was probably caused by the increase in ITT concentration. The asterisk indicates a peak exhibiting the same retention time as 2-HP. (C) GC-ToF-MS chromatogram of samples obtained from cultivation of indicated strains with the itaconate/ITT/2-HP mixture. Total ion current (TIC) is shown. The asterisk marks an unknown molecule that exhibited the same mass and MS-spectrum as 2-HP but showed a different retention time. This compound was probably detected *via* HPLC, showing the same retention time as 2-HP. (D) Proposed dead-end pathway for 2-HP and ITT in *P. putida* KT2440 *attTn7::P*_*14f*_ -PA0879-0886 and *P. aeruginosa* PAO1. Rdo_PA_, encoded by PA0880, converted 2-HP to ITT. Accumulation of ITT might have caused its partial conversion to 2,4-dihydroxybutyrate (2,4-DHB), methylated ITT (Fig. S6), 3-hydroxy-3-methylbutyrate, and other unknown compounds detected by GC-ToF-MS. A detailed pathway from ITT to 2.4-DHB is shown in Fig. S5.

Growth of *P. putida* KT2440 *attTn7::P*_*14f*_ -PA0879-0886 with the itaconate/2-HP/ITT mixture containing 20 mM itaconate reached a similar final optical density compared to when 20 mM itaconate was used as sole carbon source but growth was slower with a rate of 0.15 ± 0.01 h^−1^ on the mixture, compared to 0.39 ± 0.02 h^−1^ on pure itaconate (Fig. 3, Fig. 4A). Hence, 2-HP and ITT were likely not assimilated by this strain. The absence of Ich and Ccl abolished growth with the mixture, which is in agreement with the results obtained when itaconate was used as sole carbon source (Fig. 3, Fig. 4A). However, growth of the Δ*ict* mutant (μ = 0.01 ± 0.00 h^−1^) with the itaconate/2-HP/ITT mixture was more impaired compared to growth on itaconate as sole substrate (μ = 0.11 ± 0.00 h^−1^) (Fig. S1). This might indicate inhibition of SucCD by ITT, given its structural similarity to itaconate.

The most prominent effect was caused by the absence of Rdo_PA_, which entirely abolished growth on itaconate in the presence of 2-HP and ITT (Fig. 4A). In contrast, pure itaconate was metabolized by the ΔPA0880 mutant (μ = 0.38 ± 0.02 h^−1^) (Fig. 3A). HPLC analysis of the samples from the *P. putida* KT2440 *attTn7::P*_*14f*_ -PA0879-0886 culture grown with the mixture confirmed the degradation of 2-HP, whereas the Δ*rdo*_*PA*_ mutant did not degrade 2HP, verifying that this reaction is catalyzed by Rdo_PA_ (Fig. 4B). Hence, 2-HP inhibited itaconate metabolism, and Rdo_PA_ was found to abolish this inhibition by converting 2-HP into ITT.

The detection of two HPLC peaks near the retention time of ITT led to the assumption that other products accumulated during 2-HP degradation, which were not further metabolized (Fig. 4B). Growth experiments with *P. aeruginosa* PAO1 revealed accumulation of the same degradation products (Fig. 4B). Gas chromatography time-of-flight mass spectrometry (GC-ToF-MS) was used to identify the unknown dead-end metabolites by comparing samples of *P. putida* KT2440 *attTn7::P*_*14f*_ -PA0879-0886 and the Δ*rdo*_*PA*_ mutant. A detailed list of all detected molecules is summarized in Table S3. This analysis revealed the accumulation of ITT in the sample expressing the PA0878-0886 operon (Fig. 4C, Table S3). The increased ITT concentration probably caused the observed peak shift of ITT during HPLC analysis (Fig. 4B and C). No 2-HP was detected in this sample, confirming its entire conversion to ITT. Since the accumulation of ITT by *P. putida* KT2440 *attTn7::P*_*14f*_ -PA0879-0886 did not affect growth, 2-HP alone, and not ITT, likely inhibited growth of the Δ*rdo*_*PA*_ mutant on itaconate. In both samples, a compound exhibiting the identical mass and a similar MS-spectrum but a different retention time as 2-HP was detected by GC-ToF-MS (Fig. 4C, Fig. S4, and Table S3). Since 2-HP is a chiral molecule, this compound might be the enantiomeric form of 2-HP that was found to be not degraded by Rdo_PA_. This theory is confirmed by residual peaks detected by HPLC that showed the same retention time as 2-HP in samples that expressed *rdo*_*PA*_ (Fig. 4B and C). According to Guevarra and Tabuchi, (*S*)-2-HP is produced from itaconate by *U. cynodontis* (Guevarra and Tabuchi, 1990) that would reveal Rdo_PA_ to act enantioselective on the (*S*)-form. The only other major peak detected by GC-ToF-MS was 2,4-dihydroxybutyrate (2,4-DHB), which emerged in the sample that expressed *rdo*_*PA*_, suggesting it as a product of a dead-end pathway of 2-HP degradation (Fig. 4D). 2,4-DHB was also identified in metabolomics analysis of *P. putida* DOT-T1E (Sayqal et al., 2016) as well as in human blood (Hoffmann et al., 1993) and urine (Bouatra et al., 2013) without indications for a specific function. Interestingly, 2,4-DHB was identified as an inhibitor of the eukaryotic malic enzyme that catalyzes the oxidative decarboxylation of malate to pyruvate, which is a relevant reaction for a wide range of metabolic pathways (Rognstad and Katz, 1979; Schimerlik and Cleland, 1977). So far, no native bacterial pathway is known for the production of 2,4-DHB, although a synthetic pathway was recently described from malate *via* malyl-4-phosphate and malate-4-semialdehyde (Zhu et al., 2022). Given the circumstances, it is more likely that 2,4-DHB is produced from ITT (Fig. 4D), but a mechanism of this conversion remains to be elucidated. Based on the structural similarity of ITT to citrate, we speculate that ITT could be first dehydrated by an aconitase-like hydratase to 2-(hydroxymethyl)fumaric acid. Subsequent decarboxylation by a CAD-like enzyme followed by re-hydration would result in the production of 2,4-DHB (Fig. S5).

### 2.4 Effects of (*S*)-2-hydroxyparaconate and itatartarate on microbial metabolism

The observed inhibitory effect of 2-HP on itaconate metabolism led to the question if growth with itaconate was specifically inhibited or if 2-HP is a general inhibitor of microbial metabolism. To test these hypotheses, strains were grown with a mixture containing approximately 33 mM of 2-HP and 20 mM of ITT, but no itaconate, in the presence of glucose or acetate as substrates. This mixture was produced with the engineered *Ustilago cynodontis* NBRC9727 Δ*fuz7* Δ*cyp3 P*_*etef*_-*mttA P*_*ria1*_-*ria1 P*_*etef*_-*cyp3* that was optimized for production of the itaconate derivatives (Ernst et al., manuscript in preparation).

**Fig. 5:**
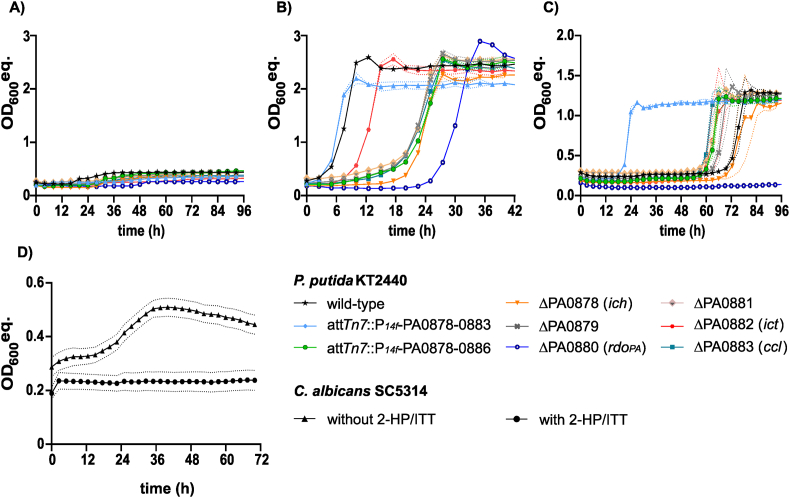
Inhibitory effect of 2-hydroxyparaconate and/or itatartarate on engineered *P. putida* strains and *C. albicans*. (A) *Pseudomonas* strains were cultivated in a Growth Profiler in 96-well microtiter plates with minimal medium containing a 2-HP/ITT mixture with approximately 33 mM 2-HP and 20 mM ITT, (B) supplemented with 15 mM glucose, (C) or 60 mM acetate. OD_600_ equivalents (OD_600_ eq.) were derived from green-values obtained from the Growth Profiler using a calibration curve. Growth rates are shown in Table S1.The mean and standard deviation (SD) of three replicates is shown. (D) *C. albicans* SC5314 was grown in a Tecan plate reader in YNB medium supplemented with 244 mM acetate as sole carbon source without (triangle) and with the same mixture of 2-HP and ITT (circle). The mean values and standard error of the mean (SEM) of five replicates is shown.

Growth of *P. putida* KT2440 *attTn7::P*_*14f*_ -PA0879-0886 and its mutant strains in the presence of the 2-HP/ITT mixture without the addition of glucose or acetate did not result in growth (Fig. 5A). Hence none of the itaconate derivatives was metabolized as carbon source. Surprisingly, the engineered strain harboring the *attTn7::P*_*14f*_ -PA0879-0886 expression cassette grew slower on glucose in the presence of 2-HP and ITT (μ = 0.23 ± 0.00 h^−1^), compared to the wild-type strain (μ = 0.32 ± 0.00 h^−1^), and also showed a longer lag-phase (Fig. 5B).

Growth of the Δ*rdo*_*PA*_ mutant was even further impaired with a lag-phase of approximately 24 h and a growth rate of 0.20 ± 0.01 h^−1^. Since Rdo_PA_ was revealed to convert 2-HP into ITT, these results indicate an inhibitory effect of 2-HP under the tested conditions. Although Rdo_PA_ was not present in the Δ*rdo*_*PA*_ mutant and no homolog was identified in this strain, the mutant was still able to grow with glucose after approximately 24 h. Hence, 2-HP might be partly degraded *via* an abiotic or unspecific route, decreasing the 2-HP concentration below a critical level.

Interestingly, the *attTn7::P*_*14f*_-PA0879-0883 mutant, lacking the BSCD transporter, grew faster (μ = 0.38 h ± 0.01 h^−1^) than the *attTn7*:*P*_*14f*_ -PA0879-0886 strain (μ = 0.23 ± 0.00 h^−1^). This indicates that 2-HP and ITT are probably also imported *via* PA0884-0886, facilitating the observed inhibition of growth. Additionally, the *attTn7::P*_*14f*_ -PA0879-0883 mutant grew slightly faster than the wild-type strain (0.32 ± 0.00 h^−1^) although both strains lacked the BSCD transporter. Assuming an unspecific or leaky transport of 2-HP and ITT, as revealed for itaconate and other BSCD, the presence of Rdo_PA_ in the *attTn7::P*_*14f*_ -PA0879-0883 mutant facilitated growth on glucose in the presence of 2-HP and ITT. Since degradation of 2-HP by Rdo_PA_ led to the accumulation of ITT, these results indicate an inhibitory effect of 2-HP during growth under the tested conditions, whereas ITT seemed not to affect growth.

Growth of all mutants that expressed the BSCD transporter was slower compared to wild-type and the *attTn7::P*_*14f*_ -PA0879-0883 mutant indicating that 2-HP and ITT are also imported by PA0884-0886. Among the mutants that expressed the BSCD-transporter, ΔPA0882 (*ict*) showed the fastest growth (μ = 0.31 h ± 0.00 h^−1^). This effect might be explained by the production of toxic CoA-intermediates of 2-HP and/or ITT catalyzed by Ict. Due to the activity of Ict towards many BSCD, this enzyme might also catalyze the CoA-activation of 2-HP and/or ITT. Such CoA-activated forms cannot be further degraded as no strain was able to grow with the 2-HP/ITT mixture, thus inhibiting growth by sequestration of coenzyme A. The absence of Ict in the ΔPA0882 (*ict*) mutant prevents production of such expected CoA-intermediates resulting in faster growth. Toxic effects of CoA-activated intermediates of the itaconate degradation pathway were indicated based on knockout mutants (Fig. S7). Since 2-HP and ITT might be CoA-activated by Ict, the previously proposed pathway for 2,4-DHB production from ITT (Fig. S5) could also be encoded by the PA0878-0883 cluster operating with the CoA-activated forms starting with ITT-CoA. In contrast to 2-HP and ITT, none of the other BSCD inhibited growth of the tested strains with glucose as carbon source (Fig. S8).

The presence of 2-HP and ITT also affected growth of the investigated strains when acetate was added as carbon source (Fig. 5C). Again, the *attTn7::P*_*14f*_ -PA0879-0883 mutant lacking the BSCD transporter showed fastest growth with a rate of 0.32 ± 0.01 h^−1^. Since a significantly longer lag-phase was revealed for the wild-type strain (0.22 ± 0.01 h^−1^), also lacking the BSCD transporter, the presence of the PA0878-0883 cluster and thus Rdo_PA_ facilitated growth. Hence, the inhibitory effect of 2-HP on bacterial growth was likely stronger under acetate-degrading conditions compared to when glucose was used as carbon source. In addition to this, the Δ*rdo*_*PA*_ mutant did not grow with acetate after incubation of 96 h, confirming the strong inhibitory effect of 2-HP. This inhibitory effect of 2-HP under acetate-degrading conditions could also be confirmed for the pathogenic yeast *Candida albicans* that does not harbor an Rdo_PA_/Rdo1 homolog. When the 2-HP and ITT mixture was added to the yeast nitrogen base (YNB) medium supplemented with acetate as carbon source, growth of *C. albicans* was inhibited for at least 72 h (Fig. 5D). In addition to 2-HP, several other BSCD also showed varying inhibitory effects on the investigated strains under acetate-degrading conditions (Fig. S8). Addition of 20 mM mesaconate or citraconate most strongly inhibit the growth, while itaconate at this concentration has little effect. The expression of the PA0884-0886 transporter increased the inhibitory effect of mesaconate and citraconate, as was observed for 2-HP. Interestingly, immunoregulatory effects were recently described for both mesaconate and citraconate (McGettrick and O’Neill, 2022).

Overall, the data on BSCD and 2-HP metabolism indicate an evolutionary arms race between (pathogenic) microorganisms and their host, or competing microbial species in ecological niches. The production of itaconate was found to inhibit the glyoxylate shunt that is essential for pathogenic bacteria to survive in the host environment (Hillier and Charnetzky, 1981). Adaption of pathogenic or competing species to itaconate inhibition through its metabolism might have led to the emergence of the novel itaconate-derived compounds inhibiting growth of the adapted bacteria. Production of 2-HP by *Ustilago* ssp. might be a strategy to combat species capable of metabolizing itaconate. However, the exact mechanism of action of the discovered anti-bacterial and anti-fungal properties of 2-HP still needs to be identified. Currently, the production of 2-HP and ITT as pure compounds is investigated that will enable such studies.

## 3 Conclusions and outlook

Bio-upcycling of BSCD is of great importance as their usage as bio-based building blocks for plastics production is increasing. To enable bio-upcycling, funneling of these compounds into the central metabolism of suitable upcycling strains is required. Heterologous expression of the PA0878-0886 cluster resulted in an engineered strain of *P. putida* KT2440 able to rapidly metabolize a variety of BSCD. Besides native PHA production, future implementations of biosynthetic pathways in the constructed strain will potentially enable production of further value-added compounds from polymers containing BSCD as building blocks and thus allow bio-upcycling of plastic waste.

Besides their use as plastic monomers, many BSDC such as itaconate or mesaconate exhibit anti-bacterial properties or trigger immunomodulatory effects in macrophages. The characterization of the PA0878-0886 gene cluster revealed the metabolic pathways of such relevant BSCD. The profound knowledge on the metabolic pathways gained in this study is a basis for upcoming research to identify potential drug targets against pathogenic species. Additionally, this study revealed a link of BSCD metabolism to fungal secondary metabolite resistance stressing the versatile lifestyle of *Pseudomonas* as both environmental and pathogenic organism, and highlighting the potential of 2-HP as drug candidate.

All in all, this study extends knowledge on BSCD metabolism in *P. aeruginosa*, it uncovers an intriguing link to fungal interactions of this bacterium in the environment, and provides an engineered strain of *P. putida* enabling the bio-upcycling of BSCD-based plastics. It further expands the solution space of catalytic and transport activities that can be exploited and engineered for synthetic pathway development.

## 4 Materials and methods

### 4.1 Strains and culture conditions

All chemicals used in this study were obtained from Sigma-Aldrich (St. Louis, MO, USA), Carl Roth (Karlsruhe, Germany), or Merck (Darmstadt, Germany) unless stated otherwise. All bacterial strains used in this study are listed in Table 1. *P. putida* KT2440 strains and *P. aeruginosa* PAO1 were cultivated in 5-fold buffered (19.40 g L^−1^ K_2_HPO_4_ and 8.15 g L^−1^ NaH_2_PO_4_) mineral salt medium (MSM) (Wierckx et al., 2005). Pre-cultures contained 20 mM glucose and 2 mL of culture volume was cultivated in 14 mL culture tubes (Greiner bio-one, Frickenhausen, Germany) in a Multitron shaker (Infors, Bottmingen, Switzerland) at 30 °C and 180 rpm shaking speed. For cultivations with dicarboxylates as substrates, stock solutions of 500 mM itaconic acid, (*S*)-citramalic acid, (*R*)-citramalic acid, (*S*)-methylsuccinic acid (Ambeed, Arlington Hts, IL, USA), (*R*)-methylsuccinic acid, and citraconic acid were diluted in MSM to reach a final concentration of 20 mM. Due to the lower solubility of mesaconic acid, a 200 mM stock solution was prepared for the substrate that was diluted in MSM to reach a final concentration of 20 mM. For online growth detection, a Growth Profiler 960 (Enzyscreen, Heemstede, The Netherlands) was used. This device analyses cultures in microtiter plates with transparent bottoms by image analysis. The resulting green-values (G-values, based on green pixel counts) correlate with the optical density of a cell culture. These G-values were converted into OD600 equivalents using a calibration curve for *P. putida*. Main cultures were cultivated in 96-well plates (CR1496dg) with a volume of 200 μL at 30 °C and 225 rpm shaking speed with an amplitude of 50 mm. Pictures for growth analysis were taken every 30 min.

**Table 1:** Strains used and generated in this study.

Strains	Description	Reference
*P. aeruginosa* PAO1	Wild-type, originating from Dieter Haas laboratory (Lausanne, CH)	Holloway et al. (1979)
***P. putida* strains**		
KT2440	Strain derived of *P. putida* mt-2 cured of the pWW0 plasmid	Bagdasarian et al. (1981)
*attTn7*::*P*_*14f*_ -PA0878-0883	*P. putida* KT2440 with genomic integration of the PA0878-0883 cluster under control of the constitutive *P*_*14f*_ -promoter	This study
*attTn7*::*P*_*14f*_ -PA0878-0886	*P. putida* KT2440 with genomic integration of the PA0878-0886 cluster under control of the constitutive *P*_*14f*_ -promoter	This study
ΔPA0878	*P. putida* KT2440 *attTn7*::*P*_*14f*_ -PA0878-0886 with deletion of PA0878	This study
ΔPA0879-0881	*P. putida* KT2440 *attTn7*::*P*_*14f*_ -PA0878-0886 with deletion of PA0879-0881	This study
ΔPA0879	*P. putida* KT2440 *attTn7*::*P*_*14f*_ -PA0878-0886 with deletion of PA0879	This study
ΔPA0880	*P. putida* KT2440 *attTn7*::*P*_*14f*_ -PA0878-0886 with deletion of PA0880	This study
ΔPA0881	*P. putida* KT2440 *attTn7*::*P*_*14f*_ -PA0878-0886 with deletion of PA0881	This study
ΔPA0882	*P. putida* KT2440 *attTn7*::*P*_*14f*_ -PA0878-0886 with deletion of PA0882	This study
ΔPA0883	*P. putida* KT2440 *attTn7*::*P*_*14f*_ -PA0878-0886 with deletion of PA0883	This study
ΔPP_0897	*P. putida* KT2440 *attTn7*::*P*_*14f*_ -PA0878-0886 with deletion of PP_0897	This study
***E. coli* strains**		
HB101 pRK2013	*F*^*−*^*mcrB mrr hsdS20*(*rB*^*−*^*mB*^*−*^) *recA13 leuB6 ara-14 proA2 lacY1 galK2 xyl-5 mtl-1 rpsL20*(Sm^R^) *gln V44λ*^*−*^	Boyer and Roulland-Dussoix (1969)
PIR2	F^−^ Δ*lac169 rpoS* (Am) *robA1 creC510 hsdR514 endA reacA1 uidA* (Δ*Mlui*)::*pir*	Life Technologies
DH5α λpir pTNS1	*endA1 hsdR17 glnV44* (= *supE44*) *thi-1 recA1 gyrA96 relA1* φ*80dlac*Δ(*lacZ*)*M15* Δ(*lacZYA*-*argF*)*U169 zdg*-*232*::Tn10 *uidA*::*pir*+	de Lorenzo lab
**Fungal strains**		
*Ustilago cynodontis* NRBC 9727 Δ*fuz*^*7*^	Δ*fuz*^*7*^	Hosseinpour Tehrani et al. (2019)
*Ustilago cynodontis* NBRC9727 Δ*fuz7* Δ*cyp3* P_etef_-*mttA* P_*ria1*_-*ria1* P_etef_-*cyp3*	Δ*fuz*^*7*^ Δ*cyp3* P_etef_-*mttA* P_*ria1*_*ria1* P_etef_-*cyp3*	Ernst et al.., Manuscript in preparation
*Candida albicans* SC5314	Wild-type clinical isolate	Laboratory: Wilson RB, Davis D, Enloe BM, Mitchell AP

For production of the itaconate, 2-HP, and ITT mixture, *Ustilago cynodontis* NRBC 9727 Δ*fuz*^*7*^ was used (Hosseinpour Tehrani et al., 2019). A further modified strain was used for the production of the 2-HP and ITT mixture not containing itaconate anymore (Ernst et al., manuscript in preparation). For producing the mixtures, pre-cultures of the *U. cynodontis* strains were grown in yeast extract (20 g L^−1^) peptone (20 g L^−1^) sucrose (20 g L^−1^) (YEPS) medium at 30 °C and 250 rpm for 48 h. Main cultures were performed in modified Tabuchi medium (MTM) according to Geiser et al. (2014). The final MTM contained 100 mM 2-(N-morpholino)ethanesulfonic acid (MES; pH adjusted to 6.5 with NaOH), 0.8 g L^−1^ NH_4_Cl, 0.2 g L^−1^ MgSO_4_·7H_2_O, 0.01 g L^−1^ FeSO_4_·7H_2_O, 0.5 g L^−1^ KH_2_PO_4_, 1 mL L^−1^ vitamin solution, and 1 mL L^−1^ trace element solution. The vitamin solution contained (per liter) 0.05 g D-biotin, 1 g D-calcium panthotenate, 1 g nicotinic acid, 25 g myo-inositol, 1 g thiamine hydrochloride, 1 g pyridoxol hydrochloride, and 0.2 g *para*-aminobenzoic acid. The trace element solution contained (per liter) 1.5 g EDTA, 0.45 g ZnSO_4_·7H_2_O, 0.10 g MnCl_2_·4H_2_O, 0.03 g CoCl_2_·6H_2_O, 0.03 g CuSO_4_·5H_2_O, 0.04 g Na_2_MoO_4_·2H_2_O, 0.45 g CaCl_2_·2H_2_O, 0.3 g FeSO_4_·7H_2_O, 0.10 g H_3_BO_3_, and 0.01 g KI. Shaking cultures were performed in 500 mL shake flasks with a filling volume of 50 mL and were cultivated at 30 °C and 250 rpm with a humidity of 80% for 5 days. After that, cells were harvested for 20 min at 7000×*g*. The supernatant was diluted 2-fold with ultrapure H_2_O and the pH was adjusted to pH 6.7 using NaOH. After that, the mixture was filtered through a 0.22 μm PES syringe filter and all components for 5-fold buffered MSM were added, resulting in the final growth medium. When indicated, 15 mM glucose or 60 mM acetate were added as additional carbon sources to this mixture. *C. albicans* SC5314 (wild-type strain) was grown in yeast nitrogen base (YNB) medium supplemented with 244 mM acetate. For testing the inhibitory effects of 2-HP and ITT, the mixture was diluted to contain the same 2-HP and ITT concentrations as for experiments with *P. putida* KT2440, i.e., approximately 33 mM of 2-HP and 20 mM ITT. *C. albicans* SC5314 was grown in YNB medium and a final acetate concentration of 244 mM as carbon source was used. Measurements were performed in a Tecan infinite M plex reader and the optical density at 600 nm was analyzed.

### 4.2 Plasmid cloning and strain engineering

Genomic DNA of *P. aeruginosa* PAO1 was isolated using the Monarch® Genomic DNA Purification Kit (New England Biolabs, Ipswich, MA, USA). Primers were ordered as unmodified DNA oligonucleotides from Eurofins Genomics (Ebersberg, Germany). DNA fragments were obtained by PCR using the Q5® High-Fidelity 2 × master mix as DNA Polymerase (New England Biolabs, Ipswich, MA, USA). Plasmids were assembled by Gibson assembly (Gibson et al., 2009) using the NEBuilder HiFi DNA Assembly Master Mix (New England Biolabs). Detailed information about the oligonucleotides and plasmids used in this study are listed in Table 2 and Table 3, respectively. For the transformation of assembled DNA fragments and plasmids into competent *E. coli* cells, a heat shock protocol was used (Hanahan, 1983). The integration of heterologous constructs from *P. aeruginosa* PAO1 into the *attTn7*-site of *P. putida* KT2440 was performed by patch mating. For this, the *E. coli* PIR2 donor strain holding the respective pBG14f_FRT_Kan plasmid, the helper strain *E. coli* HB101 pRK2013, the transposase-providing *E. coli* HD5α λpir pTNS1, and the recipient *P. putida* KT2440 were used. For the generating the ΔPP_0897 mutant of *P. putida* KT2440 *attTn7::P*_*14f*_ –PA0878-0886 the I-SceI-based system (Martínez-García and de Lorenzo, 2011) was used according to the streamlined protocol (Wynands et al., 2018). The 500–600 bp up- and downstream flanking regions (TS1 and TS2) of PP_0897 were integrated into the suicide delivery vector pSEVA512S. Positive clones were iteratively inoculated in LB medium to cure the strain from pSW-2.

**Table 2:** Oligonucleotides used in this study.

Primer	Sequence 5′-3′	Template/Purpose
JDW001	GAATTCGAGCTCGGTACC	pBG14f_FRT_Kan backbone
JDW002	TAGAAAACCTCCTTAGCATG	pBG14f_FRT_Kan backbone
JDW003	CATGCTAAGGAGGTTTTCTAATGAGTGAGTCCGCTTTCGCCC	PA0878 fw cluster
JDW004	CGGGTACCGAGCTCGAATTCTCAGCCACCCTCCCCGGC	PA0883 rv cluster
JDW005	GTTCGGTTGATCAGTCGAATTCCACGTC	PA0878 rv for ΔPA0879-0881
JDW006	ATTCGACTGATCAACCGAACCAGCCGCACC	PA0882 fw for ΔPA0879-0881
JDW007	TCAGCCACCCTCCCCGGC	PA0883 rv
JDW009	CGGGTACCGAGCTCGAATTCTCAGCTCATGCCCAGCAG	PA0886 rv cluster
JDW010	GGGTAGTCGGTATGCAAATG	Mapping *Tn7* insertion PP_5408 fw
JDW011	TAGACGATGTCGTGCTCTTC	Mapping *Tn7* insertion PA0878 rv
JDW037	GGCTCCCGCTTCAGTCGAATTCCACGTC	PA0878 rv for ΔPA0879
JDW038	ATTCGACTGAAGCGGGAGCCGCCCATGC	PA0880 fw for ΔPA0879
JDW039	GGCACGCGGCTCAGAACGAGCGCGGCAG	PA0879 rv for ΔPA0880
JDW040	CTCGTTCTGAGCCGCGTGCCTCGGGACA	PA0881 fw for ΔPA0880
JDW041	GTTCGGTTGACTACAGCGGGTTGCTCAG	PA0880 rv for ΔPA0881
JDW042	CCCGCTGTAGTCAACCGAACCAGCCGCA	PA0882 fw for ΔPA0881
JDW043	GTCCGCCTCCTCAGATCAGCGGCGCTAC	PA0881 rv for ΔPA0882
JDW044	GCTGATCTGAGGAGGCGGACATGAACCG	PA0883 fw for ΔPA0882
JDW047	TCGAACGCGTTCATACCGTTCCGGTCGC	PA0882 rv for ΔPA0883
JDW048	AACGGTATGAACGCGTTCGACAGACGCG	PA0884 fw for ΔPA0883
JDW050	GAGGATCCCCGGGTACCG	pSEVA512S backbone
JDW051	AGAGTCGACCTGCAGGC	pSEVA512S backbone
JDW052	ATGCCTGCAGGTCGACTCTAGCAACCGCAGGCGCAACA	PP_0897 TS1 fw
JDW053	TTACATCAGGGCAGCGCGCTCCTCTTAAAG	PP_0897 TS1 rv
JDW054	AGCGCGCTGCCCTGATGTAACTGCGGCGGC	PP_0897 TS2 fw
JDW055	CTCGGTACCCGGGGATCCTCGCAGGCCAGGGGCTGCAG	PP_0897 TS2 rv

**Table 3:** Plasmids used in this study.

Plasmids	Description	Reference
pRK2013	Km^R^, *oriV*(RK2/ColE1) -mob^+^ tra^+^	Figurski and Helinski (1979)
pTNS1	Ap^R^, *oriV*(R6K), *tnsABC*+*D* operon	Choi et al. (2005)
pBG14f_FRT_Kan	Km^R^ flanked with FRT sites, *oriV*(R6K), pBG-derived, promoter 14f, *msfGFP*	Ackermann et al. (2021)
pBG14f_FRT_Kan PA0878-0883		This study
pBG14f_FRT_Kan PA0878-0886		This study
pBG14f_FRT_Kan PA0878-0886 ΔPA0878		This study
pBG14f_FRT_Kan PA0878-0886 ΔPA0879-0881		This study
pBG14f_FRT_Kan PA0878-0886 ΔPA0879		This study
pBG14f_FRT_Kan PA0878-0886 ΔPA0880		This study
pBG14f_FRT_Kan PA0878-0886 ΔPA0881		This study
pBG14f_FRT_Kan PA0878-0886 ΔPA0882		This study
pBG14f_FRT_Kan PA0878-0886 ΔPA0883		This study
pSEVA512S	Tc^R^, *oriV*(R6K), mob^+^, *lacZα*-MCS flanked by two I-SceI sites	de Lorenzo lab
pSEVA512 S PP_0897	pSEVA512S with flanking regions TS1 and TS2 for PP_0897 knockout	This study
pSW-2	Gm^R^, *oriV*(RK2), *xylS*, Pm Ι-SceΙ (transcriptional fusion of Ι-SceΙ to Pm)	Martínez-García and de Lorenzo (2011)

### 4.3 HPLC analysis

For analyzing the mixtures of itaconate, 2-HP, and ITT, samples were taken from liquid cultivations and were filtered through an AcroPrep™ 96-well filter plate (Pall Corporation, Port Washington, NY, USA) to obtain the analytes for High-Performance Liquid Chromatography (HLPC) analysis. HPLC analysis was performed using a 1260 Infinity II HPLC equipped with a refractive index detector (RID) and a diode array detector (DAD) (Agilent, Santa Clara, California, USA). Itaconate, 2-HP, and ITT were detected using the DAD at 210 nm. Analytes were eluted using a 300 × 8 mm organic acid resin column (Metab-AAC, Isera, Düren, Germany) together with a 40 × 8 mm organic acid resin pre-column with 5 mM H_2_SO_4_ as mobile phase at a constant flow rate of 0.6 mL min^−1^ at 40 °C.

### 4.4 GC-ToF-MS analysis

For sample preparation, cultures were filtered through an AcroPrep™ 96-well filter plate to obtain cell-free filtrates (Pall Corporation, Port Washington, NY, USA). Aliquots of 130 μL were shock frozen in liquid nitrogen and stored at −20 °C. Prior to analysis, samples were lyophilized overnight in a Christ LT-105 freeze drier (Martin Christ Gefriertrocknungsanlagen, Osterode am Harz, Germany). Two-step derivatization of the samples and GC-ToF-MS analysis was performed as described before by Paczia et al. (2012) using a L-PAL3-S15 liquid auto sampler coupled to a LECO GCxGC HRT+ 4D high resolution time of flight mass spectrometer (LECO, Mönchengladbach, Germany). To identify known metabolites a baseline noise corrected fragmentation pattern together with the corresponding current RI value (Retention time Index) was compared to our in-house accurate m/z database JuPoD, and the commercial nominal m/z database NIST20 (National Institute of Standards and Technology, USA). Unknown peaks were identified by a virtual reconstruction of the derivatized metabolite structure *via* the measured baseline noise corrected accurate mass m/z fragment pattern in comparison to an accurate m/z fragment register inside the JuPoD main library and were subsequently verified by virtual derivatization and fragmentation of the predicted structure.

### 4.5 *In silico* tools

For the prediction of operons the operon-mapper was used and “predicted operons” was set as output option (Taboada et al., 2018). Protein structures were predicted using ColabFold (Mirdita et al., 2021). ColabFold is an optimized version of AlphaFold2 (Jumper et al., 2021) using MMseqs2 allowing faster predictions of protein structures maintaining a high accuracy of the predictions. The platform is accessible *via* Google Colaboratory. Structure-based identity searches of predicted protein structures were performed using Distance Matrix Alignment (DALI) that aligned the predicted structure to entries of the Protein Data Bank (PDB) (Holm, 2020). The structures were aligned to all entries of the PDB.

## CRediT author statement

**J. de Witt:** Investigation, Writing – Original draft, Writing – Review and editing, Visualization, Validation **P. Ernst:** Investigation **J. Gätgens:** Investigation, Resources **S. Noack:** Conceptualization, Resources, Writing – Review and editing **D. Hiller:** Investigation **B. Wynands:** Writing – Review and editing, Conceptualization **N. Wierckx:** Conceptualization, Supervision, Funding acquisition, Writing – Review and editing.

## Data sharing plans

All relevant data is presented in the manuscript or the supplementary information. Further supporting raw datasets can be provided through the Jülich Data repository at https://data.fz-juelich.de/if needed.

## Declaration of competing interest

The authors declare no competing interest.

## Data Availability

Data will be made available on request.
