# Supplementary material for: Characterization and engineering of branched short-chain dicarboxylate metabolism in Pseudomonas reveals resistance to fungal 2-hydroxyparaconate
Source: Metab Eng. 2023 Jan;75:205–16. doi: 10.1016/j.ymben.2022.12.008 (PMC9875883; doi:10.1016/j.ymben.2022.12.008)
Supplement: Multimedia component 1 [file mmc1.docx]

# Supplementary material to:

# Characterization and engineering of branched short-chain dicarboxylate metabolism in *Pseudomonas* reveals resistance to fungal 2-hydroxyparaconate

Jan de Witt^1^, Philipp Ernst^1^, Jochem Gätgens^1^, Stephan Noack^1^, Davina Hiller^2^, Benedikt Wynands^1^, Nick Wierckx^1*^

^1^ Institute of Bio- and Geosciences IBG-1: Biotechnology, Forschungszentrum Jülich, Jülich, Germany

^2^ Institut für Mikrobiologie, Technische Universität Braunschweig, Germany

<https://doi.org/10.1016/j.ymben.2022.12.008>

*Corresponding author:
Nick Wierckx
E-mail: [n.wierckx@fz-juelich.de](mailto:n.wierckx@fz-juelich.de)


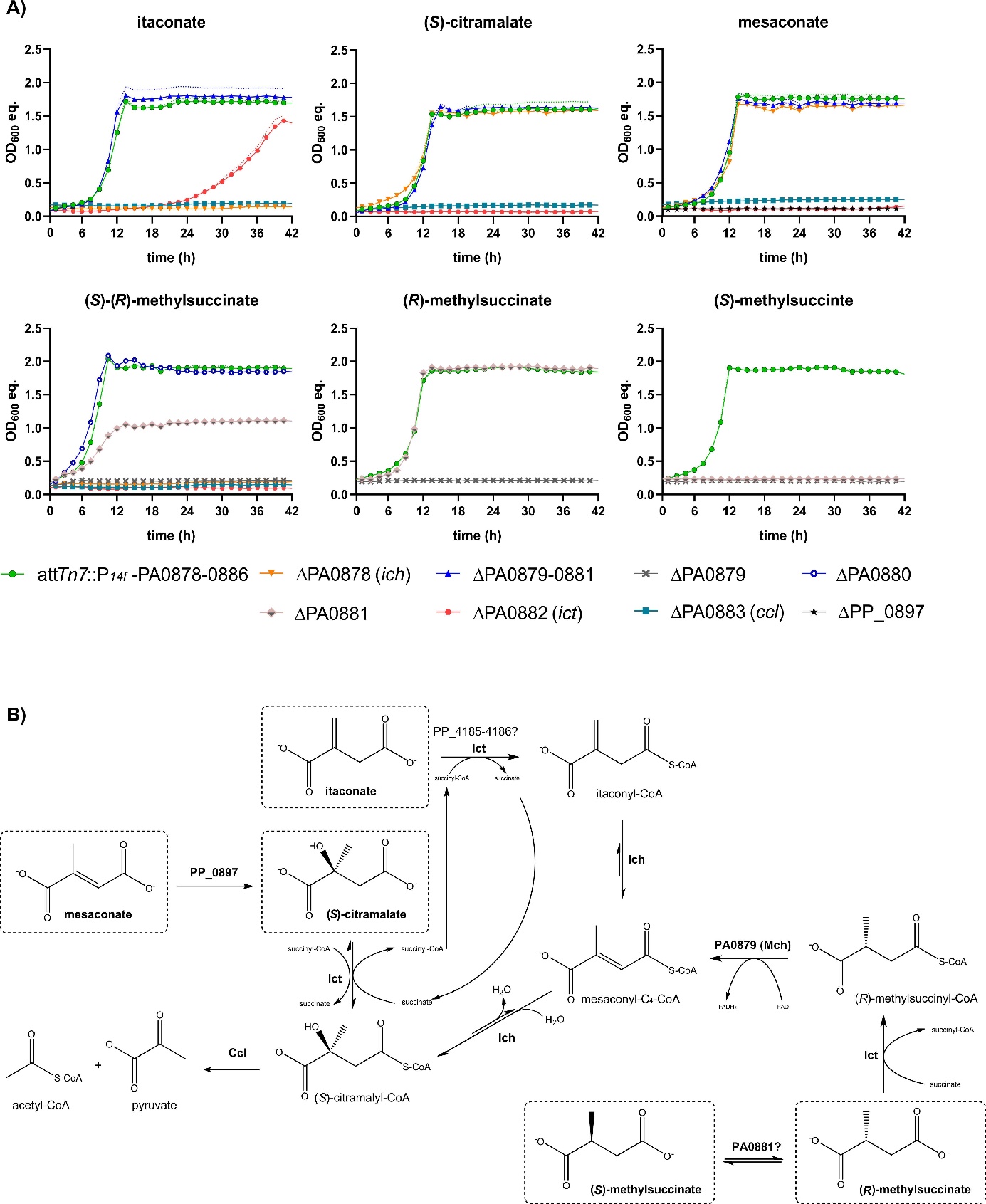


Figure S1. Growth of *P. putida* KT2440 strains on indicated BSCD. Strains were grown in a Growth Profiler in 96-well microtiter plates with MSM supplemented with 20 mM of the indicated substrate. OD_600_ equivalents (OD_600_ eq.) were derived from green-values obtained from the Growth Profiler using a calibration curve. The mean values with standard deviation (SD) of three replicates are shown. Growth rates are displayed in Table S1.


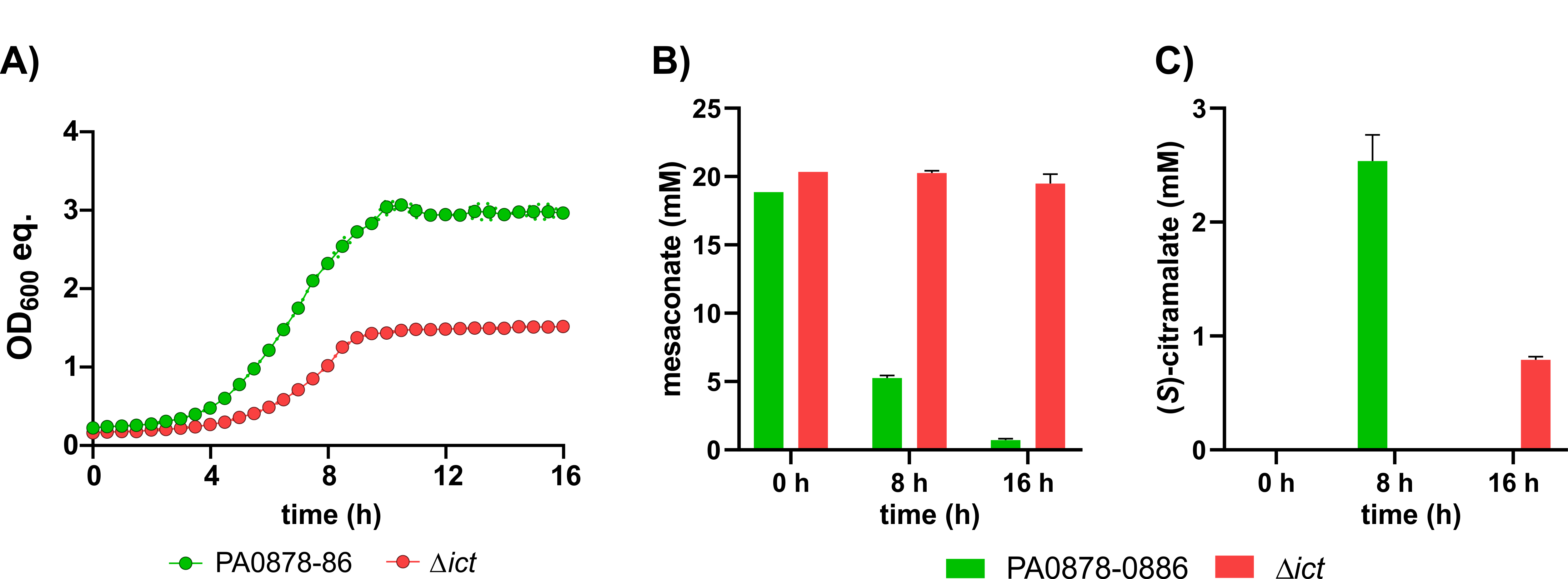


Figure S2. Growth of *P. putida* KT2440 *attTn7::P_14f_* -PA0878-86 and Δ*ict* mutant with mesaconate and glucose as mixed substrates. Strain were cultivated with 15 mM glucose and 20 mM mesaconate in a Growth Profiler in 96-well microtiter plates with MSM supplemented with 20 mM of the indicated substrate. (A) OD_600_ equivalents (OD_600_ eq.) were derived from green-values obtained from the Growth Profiler using a calibration curve. The mean values with standard deviation (SD) of three replicates are shown. The Δ*ict* mutant was not able to metabolize mesaconate (see Figure S1). HPLC analysis of culture supernatants for quantifying mesaconate (B) and (*S*)-citramalate (C) concentrations.


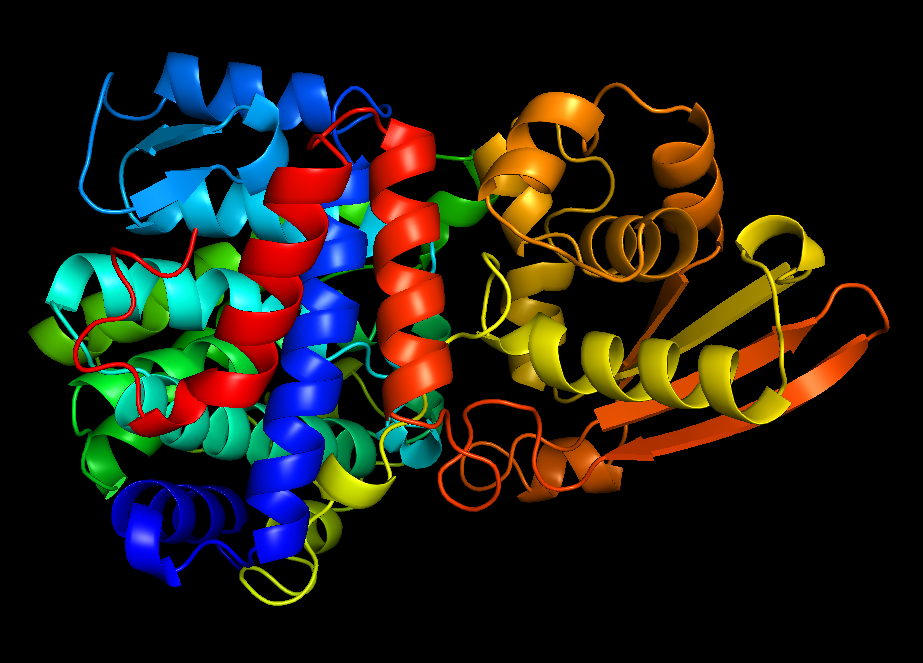


Figure S3. Predicted structure of PA0881. Structure was predicted using ColabFold (Mirdita et al., 2021) with N to C rainbow color code.


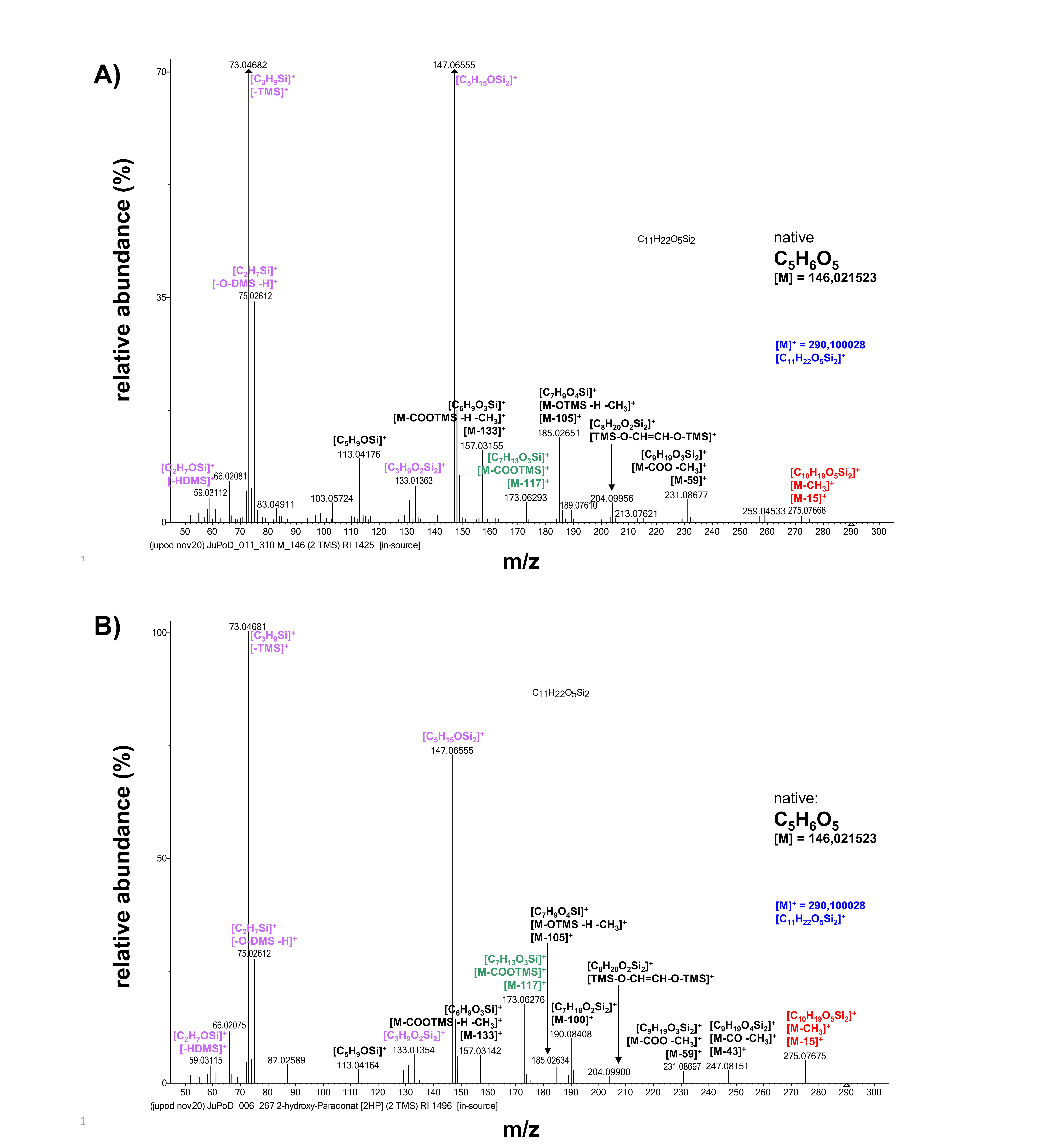


Figure S4. m/z-spectrum of 2-HP (A) and 2-HP* (B). Both compounds exhibit the same mass and similar MS-spectra. Hence, they might be (*S*)-(*R*)-isomers. Only one form was found to be cleaved by Rdo_PA_.


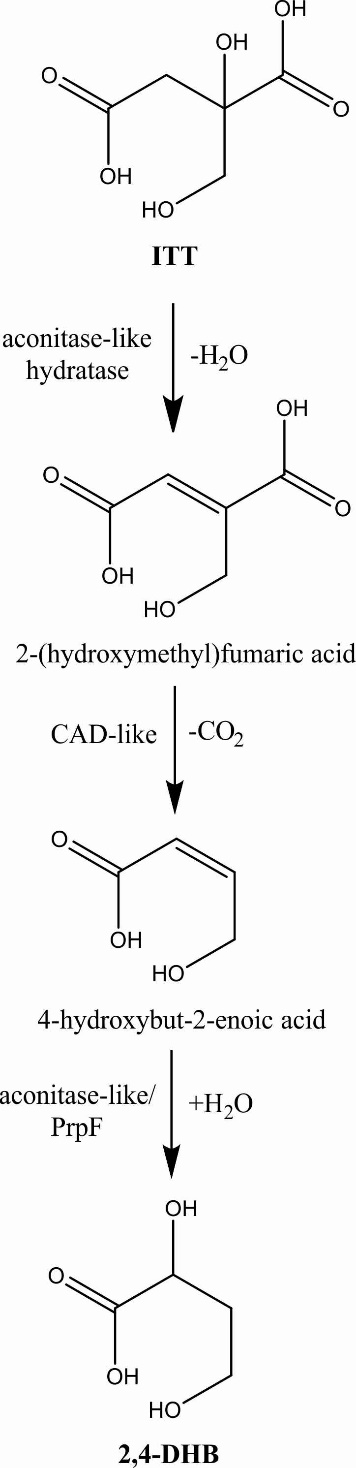


Figure S5: Proposed conversion of ITT to 2,4-DHB. Since activity of Ict towards ITT was indicated, this pathway might proceed *via* the CoA-activated forms starting with ITT-CoA.


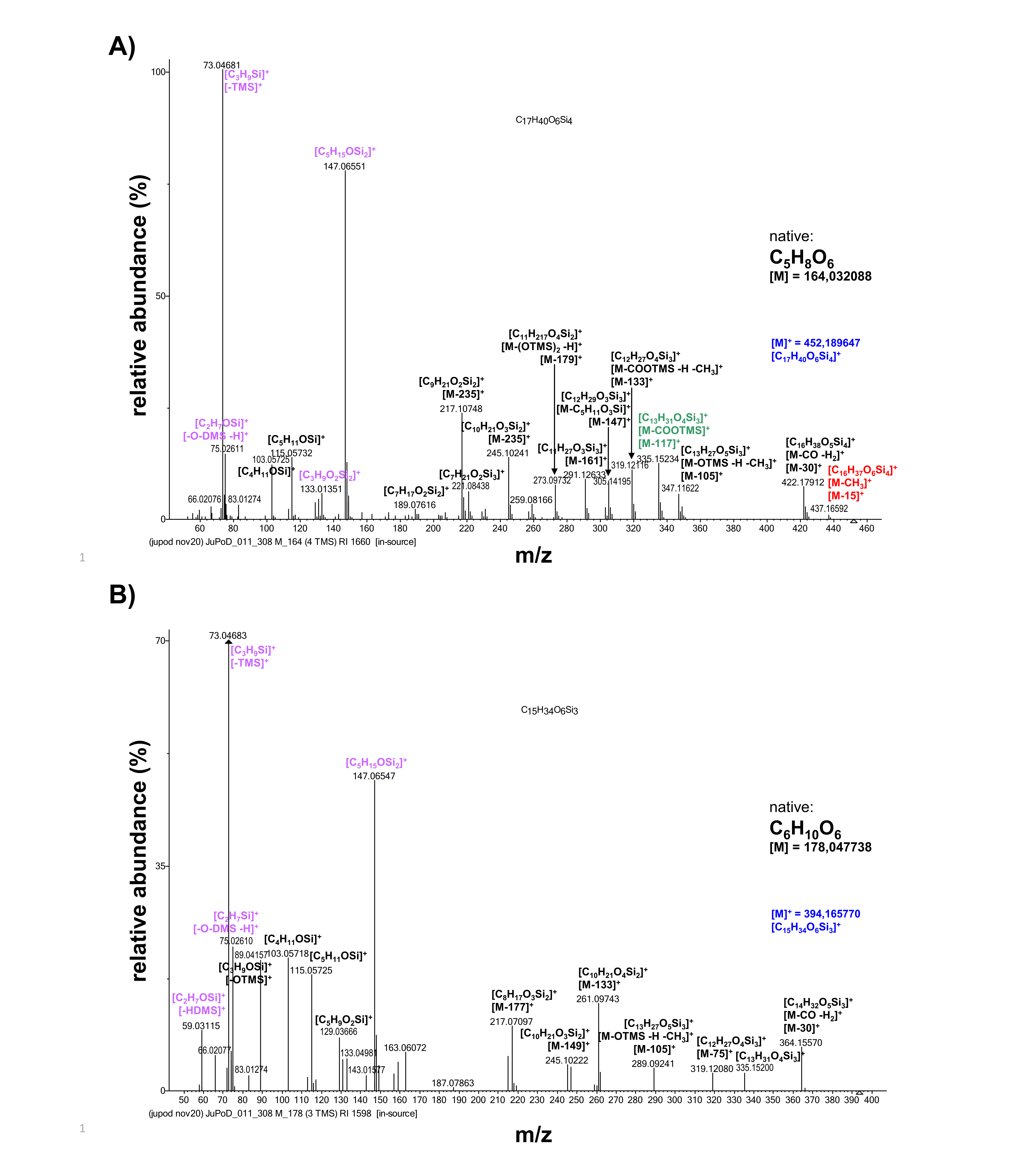


Figure S6. m/z-spectrum of ITT (A) and probably methylated ITT (B).


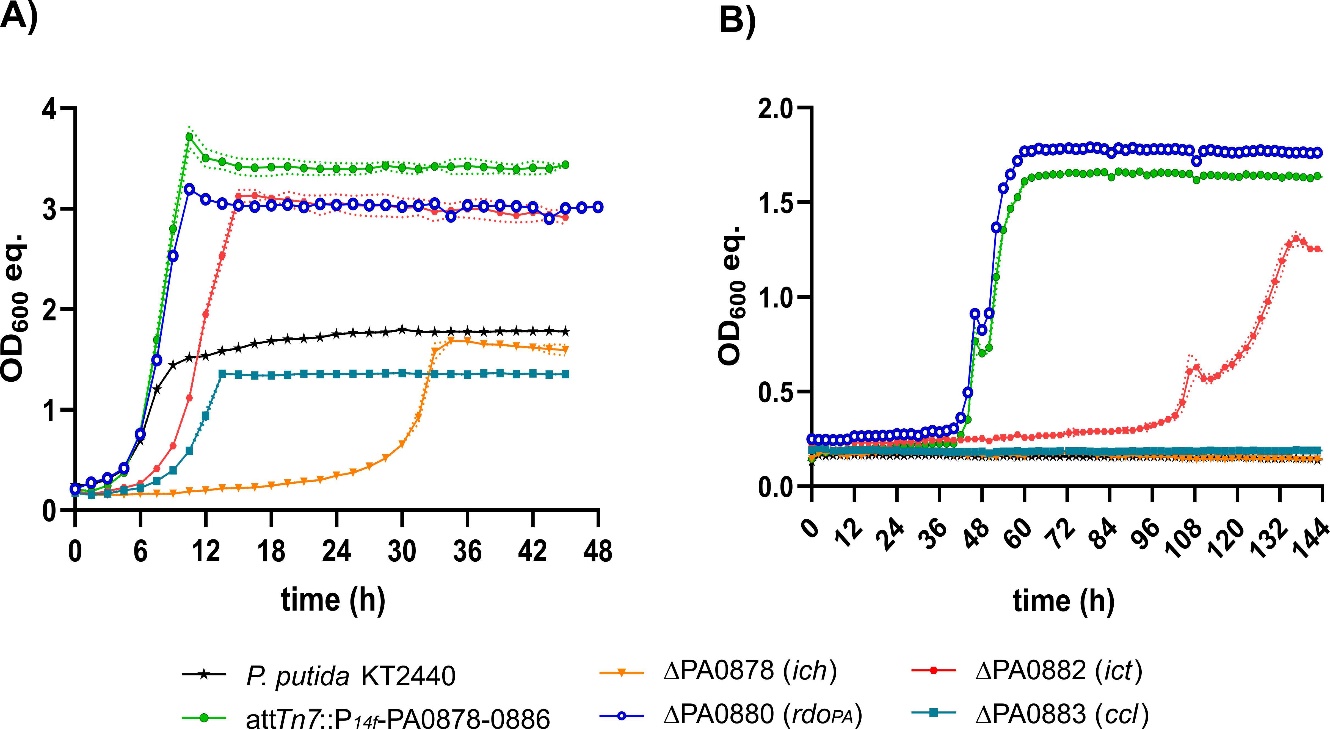


Figure S7. Effect of CoA-intermediates originating from incomplete itaconate degradation on glucose and acetate metabolism. Strains were grown in the presence of 20 mM itaconate and 15 mM glucose (A) or 60 mM acetate (B). The following accumulation products are expected for the tested mutants that impaired growth on the substrates: itaconyl-CoA (∆Ich), (*S*)-citramalyl-CoA (∆Ccl). Due to the putative side activity of the native SucCD on itaconate, the ∆Ict mutant showed delayed growth but could metabolize itaconate. OD_600_ equivalents (OD_600_ eq.) were derived from green-values obtained from the Growth Profiler using a calibration curve. The mean values with standard deviation (SD) of three replicates are shown. Growth rates are shown in Table S1.


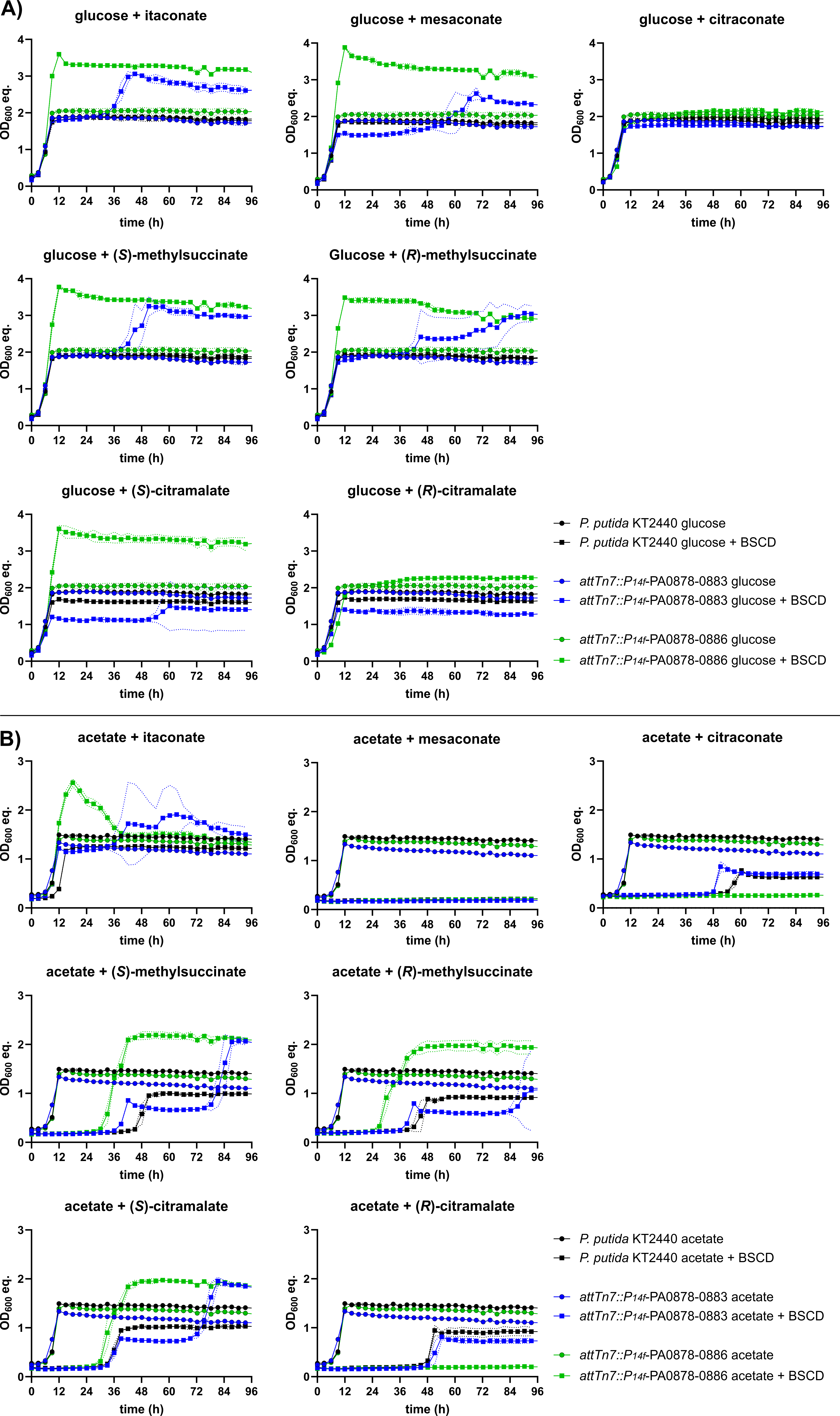


**Figure S8. Toxicity of branched short-chain dicarboxylates for *P. putida* strains under glucose- and acetate-degrading conditions.** Strains were cultivated in mineral salts medium with 15 mM glucose (A) or 60 mM acetate (B) supplemented with 20 mM of the indicated BSCD. OD_600_ equivalents (OD_600_ eq.) were derived from green-values obtained from the Growth Profiler using a calibration curve. The mean values with standard deviation (SD) of three replicates are shown. Growth rates are shown in Table S1.

Table S1. Growth rates of *P. putida* strains used in this study.

| **Figure 2** | | | |
| --- | --- | --- | --- |
| **Strain** | **substrate** | **µ (h^-1^)** | **SD** |
| *P. putida* KT2440 attTn7::P_14f_*PA0878-0883* | itaconate | 0.11 | 0.00 |
| *P. putida* KT2440 attTn7::P_14f_*PA0878-0886* | itaconate | 0.39 | 0.02 |
| *P. putida* KT2440 attTn7::P_14f_*PA0878-0883* | mesaconate | 0.04 | 0.00 |
| *P. putida* KT2440 attTn7::P_14f_*PA0878-0886* | mesaconate | 0.42 | 0.02 |
| *P. putida* KT2440 attTn7::P_14f_*PA0878-0883* | (*S*)-citramalate | 0.01 | 0.00 |
| *P. putida* KT2440 attTn7::P_14f_*PA0878-0886* | (*S*)-citramalate | 0.40 | 0.01 |
| *P. putida* KT2440 attTn7::P_14f_*PA0878-0883* | (*R*)-citramalate | no growth | - |
| *P. putida* KT2440 attTn7::P_14f_*PA0878-0886* | (*R*)-citramalate | no growth | - |
| *P. putida* KT2440 attTn7::P_14f_*PA0878-0883* | citraconate | no growth | - |
| *P. putida* KT2440 attTn7::P_14f_*PA0878-0886* | citraconate | no growth | - |
| *P. putida* KT2440 attTn7::P_14f_*PA0878-0883* | (*S*)-(*R*)-methylsuccinate | 0.02 | 0.00 |
| *P. putida* KT2440 attTn7::P_14f_*PA0878-0886* | (*S*)-(*R*)-methylsuccinate | 0.40 | 0.02 |
|  |  |  |  |
| **Figure 4** | | | |
| **Strain** | **substrate** | **µ (h^-1^)** | **SD** |
| *P. putida* KT2440 | ITA, ITT, 2-HP | no growth | - |
| *P. putida* KT2440 attTn7::P_14f_*PA0878-0883* | ITA, ITT, 2-HP | 0.12 | 0.00 |
| *P. putida* KT2440 attTn7::P_14f_*PA0878-0886* | ITA, ITT, 2-HP | 0.15 | 0.01 |
| ΔPA0878 (*ich*) | ITA, ITT, 2-HP | no growth | - |
| ΔPA0879 | ITA, ITT, 2-HP | 0.13 | 0.01 |
| ΔPA0880 (*rdo_PA_*) | ITA, ITT, 2-HP | no growth | - |
| ΔPA0881 | ITA, ITT, 2-HP | 0.14 | 0.00 |
| ΔPA0882 (*ict*) | ITA, ITT, 2-HP | 0.01 | 0.00 |
| ΔPA0883 (*ccl*) | ITA, ITT, 2-HP | no growth | - |
|  |  |  |  |
| **Figure 5** | | | |
| **Strain** | **substrate** | **µ (h^-1^)** | **SD** |
| *P. putida* KT2440 | Glucose + ITT, 2-HP | 0.32 | 0.00 |
| *P. putida* KT2440 attTn7::P_14f_*PA0878-0883* | Glucose + ITT, 2-HP | 0.38 | 0.00 |
| *P. putida* KT2440 attTn7::P_14f_*PA0878-0886* | Glucose + ITT, 2-HP | 0.23 | 0.00 |
| ΔPA0878 (*ich*) | Glucose + ITT, 2-HP | 0.31 | 0.00 |
| ΔPA0879 | Glucose + ITT, 2-HP | 0.21 | 0.00 |
| ΔPA0880 (*rdo_PA_*) | Glucose + ITT, 2-HP | 0.20 | 0.01 |
| ΔPA0881 | Glucose + ITT, 2-HP | 0.22 | 0.00 |
| ΔPA0882 (*ict*) | Glucose + ITT, 2-HP | 0.31 | 0.00 |
| ΔPA0883 (*ccl*) | Glucose + ITT, 2-HP | 0.20 | 0.00 |
|  |  |  |  |
| *P. putida* KT2440 | Acetate + ITT, 2-HP | 0.22 | 0.01 |
| *P. putida* KT2440 attTn7::P_14f_*PA0878-0883* | Acetate + ITT, 2-HP | 0.32 | 0.01 |
| *P. putida* KT2440 attTn7::P_14f_*PA0878-0886* | Acetate + ITT, 2-HP | 0.25 | 0.00 |
| ΔPA0878 (*ich*) | Acetate + ITT, 2-HP | 0.22 | 0.02 |
| ΔPA0879 | Acetate + ITT, 2-HP | 0.23 | 0.00 |
| ΔPA0880 (*rdo_PA_*) | Acetate + ITT, 2-HP | no growth | - |
| ΔPA0881 | Acetate + ITT, 2-HP | 0.23 | 0.01 |
| ΔPA0882 (*ict*) | Acetate + ITT, 2-HP | 0.23 | 0.02 |
| ΔPA0883 (*ccl*) | Acetate + ITT, 2-HP | 0.23 | 0.01 |
|  |  |  |  |
| **Figure S1** | | | |
| **Strain** | **substrate** | **µ (h^-1^)** | **SD** |
| *P. putida* KT2440 attTn7::P_14f_*PA0878-0886* | itaconate | 0.36 | 0.01 |
| ΔPA0878 (*ich*) | itaconate | no growth | - |
| ΔPA0879-81 | itaconate | 0.41 | 0.01 |
| ΔPA0879 | itaconate | 0.38 | 0.02 |
| ΔPA0880 (*rdo_PA_*) | itaconate | 0.38 | 0.02 |
| ΔPA0881 | itaconate | 0.37 | 0.01 |
| ΔPA0882 (*ict*) | itaconate | 0.11 | 0.00 |
| ΔPA0883 (*ccl*) | itaconate | no growth | - |
| ΔPP_0897 | itaconate | 0.37 | 0.01 |
|  |  |  |  |
| *P. putida* KT2440 attTn7::P_14f_*PA0878-0886* | (*S*)-citramalate | 0.38 | 0.00 |
| ΔPA0878 (*ich*) | (*S*)-citramalate | 0.32 | 0.01 |
| ΔPA0879-81 | (*S*)-citramalate | 0.40 | 0.01 |
| ΔPA0879 | (*S*)-citramalate | 0.39 | 0.02 |
| ΔPA0880 (*rdo_PA_*) | (*S*)-citramalate | 0.37 | 0.02 |
| ΔPA0881 | (*S*)-citramalate | 0.00 |  |
| ΔPA0882 (*ict*) | (*S*)-citramalate | no growth | - |
| ΔPA0883 (*ccl*) | (*S*)-citramalate | no growth | - |
| ΔPP_0897 | (*S*)-citramalate | 0.37 | 0.01 |
|  |  |  |  |
| *P. putida* KT2440 attTn7::P_14f_*PA0878-0886* | mesaconate | 0.36 | 0.00 |
| ΔPA0878 (*ich*) | mesaconate | 0.37 | 0.01 |
| ΔPA0879-81 | mesaconate | 0.36 | 0.00 |
| ΔPA0879 | mesaconate | 0.38 | 0.02 |
| ΔPA0880 (*rdo_PA_*) | mesaconate | 0.39 | 0.01 |
| ΔPA0881 | mesaconate | 0.38 | 0.02 |
| ΔPA0882 (*ict*) | mesaconate | no growth | - |
| ΔPA0883 (*ccl*) | mesaconate | no growth | - |
| ΔPP_0897 | mesaconate | no growth | - |
|  |  |  |  |
| *P. putida* KT2440 attTn7::P_14f_*PA0878-0886* | (*S*)-(*R*)-methylsuccinate | 0.36 | 0.00 |
| ΔPA0878 (*ich*) | (*S*)-(*R*)-methylsuccinate | no growth | - |
| ΔPA0879-81 | (*S*)-(*R*)-methylsuccinate | no growth | - |
| ΔPA0879 | (*S*)-(*R*)-methylsuccinate | no growth | - |
| ΔPA0880 (*rdo_PA_*) | (*S*)-(*R*)-methylsuccinate | 0.36 | 0.01 |
| ΔPA0881 | (*S*)-(*R*)-methylsuccinate | 0.18 | 0.00 |
| ΔPA0882 (*ict*) | (*S*)-(*R*)-methylsuccinate | no growth | - |
| ΔPA0883 (*ccl*) | (*S*)-(*R*)-methylsuccinate | no growth | - |
| ΔPP_0897 | (*S*)-(*R*)-methylsuccinate | 0.36 | 0.02 |
|  |  |  |  |
| *P. putida* KT2440 attTn7::P_14f_*PA0878-0886* | (*R*)-methylsuccinate | 0.36 | 0.00 |
| ΔPA0878 (*ich*) | (*R*)-methylsuccinate | no growth | - |
| ΔPA0879-81 | (*R*)-methylsuccinate | no growth | - |
| ΔPA0879 | (*R*)-methylsuccinate | no growth | - |
| ΔPA0880 (*rdo_PA_*) | (*R*)-methylsuccinate | 0.35 | 0.01 |
| ΔPA0881 | (*R*)-methylsuccinate | 0.36 | 0.01 |
| ΔPA0882 (*ict*) | (*R*)-methylsuccinate | no growth | - |
| ΔPA0883 (*ccl*) | (*R*)-methylsuccinate | no growth | - |
| ΔPP_0897 | (*R*)-methylsuccinate | 0.37 | 0.01 |
|  |  |  |  |
| *P. putida* KT2440 attTn7::P_14f_*PA0878-0886* | (*S*)-methylsuccinate | 0.36 | 0.00 |
| ΔPA0878 (*ich*) | (*S*)-methylsuccinate | no growth | - |
| ΔPA0879-81 | (*S*)-methylsuccinate | no growth | - |
| ΔPA0879 | (*S*)-methylsuccinate | no growth | - |
| ΔPA0880 (*rdo_PA_*) | (*S*)-methylsuccinate | 0.36 | 0.02 |
| ΔPA0881 | (*S*)-methylsuccinate | no growth | - |
| ΔPA0882 (*ict*) | (*S*)-methylsuccinate | no growth | - |
| ΔPA0883 (*ccl*) | (*S*)-methylsuccinate | no growth | - |
| ΔPP_0897 | (*S*)-methylsuccinate | 0.39 | 0.01 |
|  |  |  |  |
|  |  |  |  |
| **Figure S8** | | | |
| **Strain** | **substrate** | **µ (h^-1^)** | **SD** |
| *P. putida* KT2440 | glucose | 0.38 | 0.01 |
| *P. putida* KT2440 attTn7::P_14f_*PA0878-0883* | glucose | 0.38 | 0.01 |
| *P. putida* KT2440 attTn7::P_14f_*PA0878-0886* | glucose | 0.37 | 0.01 |
| *P. putida* KT2440 | glucose + itaconate | 0.41 | 0.00 |
| *P. putida* KT2440 attTn7::P_14f_*PA0878-0883* | glucose + itaconate | 0.40 | 0.02 |
| *P. putida* KT2440 attTn7::P_14f_*PA0878-0886* | glucose + itaconate | 0.42 | 0.01 |
| *P. putida* KT2440 | glucose + mesaconate | 0.39 | 0.00 |
| *P. putida* KT2440 attTn7::P_14f_*PA0878-0883* | glucose + mesaconate | 0.39 | 0.01 |
| *P. putida* KT2440 attTn7::P_14f_*PA0878-0886* | glucose + mesaconate | 0.41 | 0.01 |
| *P. putida* KT2440 | glucose + (*S*)-citramalate | 0.39 | 0.01 |
| *P. putida* KT2440 attTn7::P_14f_*PA0878-0883* | glucose + (*S*)-citramalate | 0.40 | 0.00 |
| *P. putida* KT2440 attTn7::P_14f_*PA0878-0886* | glucose + (*S*)-citramalate | 0.41 | 0.02 |
| *P. putida* KT2440 | glucose + (*R*)-citramalate | 0.40 | 0.01 |
| *P. putida* KT2440 attTn7::P_14f_*PA0878-0883* | glucose + (*R*)-citramalate | 0.39 | 0.00 |
| *P. putida* KT2440 attTn7::P_14f_*PA0878-0886* | glucose + (*R*)-citramalate | 0.39 | 0.02 |
| *P. putida* KT2440 | glucose + (*S*)-methylsuccinate | 0.40 | 0.01 |
| *P. putida* KT2440 attTn7::P_14f_*PA0878-0883* | glucose + (*S*)-methylsuccinate | 0.39 | 0.01 |
| *P. putida* KT2440 attTn7::P_14f_*PA0878-0886* | glucose + (*S*)-methylsuccinate | 0.43 | 0.02 |
| *P. putida* KT2440 | glucose + (*R*)-methylsuccinate | 0.39 | 0.00 |
| *P. putida* KT2440 attTn7::P_14f_*PA0878-0883* | glucose + (*R*)-methylsuccinate | 0.39 | 0.01 |
| *P. putida* KT2440 attTn7::P_14f_*PA0878-0886* | glucose + (*R*)-methylsuccinate | 0.42 | 0.01 |
| *P. putida* KT2440 | glucose + citraconate | 0.39 | 0.01 |
| *P. putida* KT2440 attTn7::P_14f_*PA0878-0883* | glucose + citraconate | 0.39 | 0.01 |
| *P. putida* KT2440 attTn7::P_14f_*PA0878-0886* | glucose + citraconate | 0.39 | 0.01 |
|  |  |  |  |
| *P. putida* KT2440 | acetate | 0.41 | 0.00 |
| *P. putida* KT2440 attTn7::P_14f_*PA0878-0883* | acetate | 0.39 | 0.01 |
| *P. putida* KT2440 attTn7::P_14f_*PA0878-0886* | acetate | 0.40 | 0.01 |
| *P. putida* KT2440 | acetate + itaconate | 0.37 | 0.00 |
| *P. putida* KT2440 attTn7::P_14f_*PA0878-0883* | acetate + itaconate | 0.38 | 0.01 |
| *P. putida* KT2440 attTn7::P_14f_*PA0878-0886* | acetate + itaconate | 0.40 | 0.01 |
| *P. putida* KT2440 | acetate + mesaconate | no growth | - |
| *P. putida* KT2440 attTn7::P_14f_*PA0878-0883* | acetate + mesaconate | no growth | - |
| *P. putida* KT2440 attTn7::P_14f_*PA0878-0886* | acetate + mesaconate | no growth | - |
| *P. putida* KT2440 | acetate + (*S*)-citramalate | 0.36 | 0.01 |
| *P. putida* KT2440 attTn7::P_14f_*PA0878-0883* | acetate + (*S*)-citramalate | 0.36 | 0.01 |
| *P. putida* KT2440 attTn7::P_14f_*PA0878-0886* | acetate + (*S*)-citramalate | 0.37 | 0.01 |
| *P. putida* KT2440 | acetate + (*R*)-citramalate | 0.33 | 0.01 |
| *P. putida* KT2440 attTn7::P_14f_*PA0878-0883* | acetate + (*R*)-citramalate | 0.32 | 0.02 |
| *P. putida* KT2440 attTn7::P_14f_*PA0878-0886* | acetate + (*R*)-citramalate | no growth | - |
| *P. putida* KT2440 | acetate + (*S*)-methylsuccinate | 0.33 | 0.02 |
| *P. putida* KT2440 attTn7::P_14f_*PA0878-0883* | acetate + (*S*)-methylsuccinate | 0.31 | 0.01 |
| *P. putida* KT2440 attTn7::P_14f_*PA0878-0886* | acetate + (*S*)-methylsuccinate | 0.35 | 0.01 |
| *P. putida* KT2440 | acetate + (*R*)-methylsuccinate | 0.28 | 0.01 |
| *P. putida* KT2440 attTn7::P_14f_*PA0878-0883* | acetate + (*R*)-methylsuccinate | 0.30 | 0.01 |
| *P. putida* KT2440 attTn7::P_14f_*PA0878-0886* | acetate + (*R*)-methylsuccinate | 0.35 | 0.07 |
| *P. putida* KT2440 | acetate + citraconate | 0.26 | 0.01 |
| *P. putida* KT2440 attTn7::P_14f_*PA0878-0883* | acetate + citraconate | 0.30 | 0.06 |
| *P. putida* KT2440 attTn7::P_14f_*PA0878-0886* | acetate + citraconate | no growth | - |

Table S2. Structure-based similarity search of PA0881 using Distance Matrix Alignment (DALI). Structures are labeled with their Protein Data Bank (PDB) identifier (ID). According to Holm, a Z-score greater than 20 indicates homology of two structures (Holm, 2020). The lower the root mean square deviation of aligned C_α_ atoms (rmsd) score is, the less the average deviation in distance of C_α_ atoms in the 3D superimposition. The number of aligned C_α_ atoms is indicated by lali. IDS = iminodisuccinate, CAD = *cis*-aconitate decarboxylase, PrpD = 2-methylcitrate dehydratase.

| **PDB ID** | **Z-score** | **rmsd** | **lali** | **description** | **origin** | **reference** |
| --- | --- | --- | --- | --- | --- | --- |
| 2HP3 | 52.9 | 1.9 | 435 | IDS epimerase | *Agrobacterium  tumefaciens* BY6 | (Lohkamp et al., 2006) |
| 7BR9 | 49.3 | 2.4 | 432 | CAD | *Mus musculus* | (Chun et al., 2020a) |
| 6R6U | 48.9 | 2.4 | 434 | CAD | *Homo sapiens* | (Chen et al., 2019) |
| 7BRA | 46.1 | 2.7 | 429 | CAD | *Bacillus subtilis* | (Chun et al., 2020b) |
| 6S62 | 41.8 | 2.6 | 432 | PrpD | *Pseudomonas aeruginosa* PAO1 | Unpublished,  [Protein Data Bank](https://www.rcsb.org/structure/6S62) |

Table S3: Compounds identified by GC-ToF-MS analysis. Retention time (R.T.) and corresponding retention index are displayed. Area percentage is shown for samples obtained from cultivation of indicated strains with the itaconate/2-HP/ITT mixture. Area percentage can be used to perform semi-quantitative analysis.

| **Compound** | **R.T. (min)** | **Retention index** | **Area % ∆PA0880** | **Area % *attTn*7::P*_14f_* – PA0878-0886** |
| --- | --- | --- | --- | --- |
| unknown | 5.20 | 951.9 | ND | 0.2 |
| 1,-hexanol | 5.56 | 982.8 | 0.4 | 0.3 |
| N-morpholin | 5.69 | 994.2 | ND | 0.1 |
| pyruvate | 6.94 | 1095.7 | 0.3 | 0.3 |
| Carbonat-methoxyamin | 7.41 | 1133.3 | 0.8 | 0.8 |
| 3-hydroxy-isovalerat | 8.24 | 1201.1 | ND | 0.2 |
| unknown | 8.51 | 1224.0 | 1.7 | 1.6 |
| phosphate | 8.95 | 1261.9 | 62.5 | 64.0 |
| unknown | 9.19 | 1283.0 | 0.5 | 1.8 |
| unknown | 9.68 | 1325.5 | ND | 0.1 |
| itaconate | 9.81 | 1336.1 | 17.3 | ND |
| unknown | 10.27 | 1376.4 | ND | 0.2 |
| 2,4-dihydroxybutyrate (2,4-DHB) | 10.52 | 1397.9 | ND | 6.4 |
| 2-hydroxyparaconate | 10.92 | 1436.0 | 9.0 | ND |
| tetradecamethyl-cyclo-Heptasiloxane | 11.07 | 1450.5 | 1.8 | 1.8 |
| citramalate | 11.19 | 1462.1 | 0.3 | ND |
| 2-hydroxyparaconate* | 11.55 | 1496.2 | 0.6 | 0.7 |
| C_6_H_10_O_6_ methylated ITT? | 12.61 | 1598.1 | ND | 0.6 |
| unknown | 12.80 | 1618.3 | 0.7 | ND |
| unknown | 13.08 | 1648.7 | ND | 0.2 |
| unknown | 13.15 | 1656.6 | ND | 0.4 |
| itatartarate | 13.23 | 1664.6 | 3.5 | 20.0 |
| unknown | 16.81 | 2080.7 | 0.3 | ND |
| unknown | 17.98 | 2234.4 | 0.5 | ND |
| unknown | 19.64 | 2474.8 | 0.2 | 0.3 |

**References**

Lohkamp, B., Bäuerle, B., Rieger, P.-G., Schneider, G., 2006. Three-dimensional Structure of Iminodisuccinate Epimerase Defines the Fold of the MmgE/PrpD Protein Family. J. Mol. Biol. 362**,** 555-566.

Chen, F., Lukat, P., Iqbal, A. A., Saile, K., Kaever, V., Heuvel, J. v. d., Blankenfeldt, W., Büssow, K., Pessler, F., 2019. Crystal structure of *cis*-aconitate decarboxylase reveals the impact of naturally occurring human mutations on itaconate synthesis. Proc. Natl. Acad. Sci. 116**,** 20644-20654.

Chun, H. L., Lee, S. Y., Kim, K.-H., Lee, C. S., Oh, T.-J., Park, H. H., 2020a. The crystal structure of mouse IRG1 suggests that *cis*-aconitate decarboxylase has an open and closed conformation. PLoS One. 15**,** e0242383.

Chun, H. L., Lee, S. Y., Lee, S. H., Lee, C. S., Park, H. H., 2020b. Enzymatic reaction mechanism of *cis*-aconitate decarboxylase based on the crystal structure of IRG1 from *Bacillus subtilis*. Sci Rep. 10**,** 11305.
